# Investigation of *Candida parapsilosis* virulence regulatory factors during host-pathogen interaction

**DOI:** 10.1038/s41598-018-19453-4

**Published:** 2018-01-22

**Authors:** Renáta Tóth, Vitor Cabral, Ernst Thuer, Flóra Bohner, Tibor Németh, Csaba Papp, Leonardo Nimrichter, Gergő Molnár, Csaba Vágvölgyi, Toni Gabaldón, Joshua D. Nosanchuk, Attila Gácser

**Affiliations:** 1 Department of Microbiology, University of Szeged, Szeged, Hungary; 2 Departments of Medicine and Microbiology and Immunology, Albert Einstein College of Medicine, New York, NY USA; 3 Centre for Genomic Regulation (CRG), Barcelona Institute of Science and Technology, Barcelona, Spain; 4 Universitat Pompeu Fabra (UPF), Barcelona, Spain; 5 Institució Catalana de Recerca i Estudis Avançats (ICREA), Barcelona, Spain; 6 Laboratório de Glicobiologia de Eucariotos, Instituto de Microbiologia Professor Paulo de Góes, Universidade Federal do Rio de Janeiro, Rio de Janeiro, Brazil

## Abstract

Invasive candidiasis is among the most life-threatening infections in patients in intensive care units. Although *Candida albicans* is the leading cause of candidaemia, the incidence of *Candida parapsilosis* infections is also rising, particularly among the neonates. Due to differences in their biology, these species employ different antifungal resistance and virulence mechanisms and also induce dissimilar immune responses. Previously, it has been suggested that core virulence effecting transcription regulators could be attractive ligands for future antifungal drugs. Although the virulence regulatory mechanisms of *C. albicans* are well studied, less is known about similar mechanisms in *C. parapsilosis*. In order to search for potential targets for future antifungal drugs against this species, we analyzed the fungal transcriptome during host-pathogen interaction using an *in vitro* infection model. Selected genes with high expression levels were further examined through their respective null mutant strains, under conditions that mimic the host environment or influence pathogenicity. As a result, we identified several mutants with relevant pathogenicity affecting phenotypes. During the study we highlight three potentially tractable signaling regulators that influence *C. parapsilosis* pathogenicity in distinct mechanisms. During infection, *CPAR2_100540* is responsible for nutrient acquisition, *CPAR2_200390* for cell wall assembly and morphology switching and *CPAR2_303700* for fungal viability.

## Introduction

Based on our rapidly expanding knowledge about the function of signaling regulators and their applicability as novel drug targets, approaches of modifying the transcriptional factors (TFs) of parasites and pathogenic species have been investigated as an alternative method to treat infections^1,2^. In addition, due to the emerging resistance of pathogenic species against different antimicrobial drugs and to the fact that drug toxicity is still a concern, applying an alternative method for drug design is appealing. So far, hundreds of virulence related transcriptional regulators have been identified and characterized in both pathogenic bacterial and fungal species, and several of these are involved in multiple virulence regulatory mechanisms^3–6^. Although the incidence of invasive fungal infections is lower than those caused by certain bacterial species, the mortality rate is comparable, particularly as the populations most impacted by invasive mycoses are immunocompromised. For example, invasive candidiasis caused by *Candida albicans* and non-*albicans Candida* (NAC) species remains one of the most common fungal infection at intensive care units, with more than 400,000 new life-threatening cases occurring annually worldwide^7^. To support the applicability of transcriptional regulators as potential targets, the pathogenic yeast *C. albicans* has regulatory processes that connect distinct mechanisms of virulence (Supplementary Fig. 1)^8–11^. Although the major pathogenicity effecting mechanisms are well understood in *C. albicans*, only a few have been studied in NAC species, such as *Candida parapsilosis*, which is often the second most frequently isolated opportunistic *Candida* species. In light of previously revealed differences in epidemiology, antifungal resistance, virulence mechanisms, and triggered immune responses between *C. parapsilosis* and *C. albicans*, an in-depth examination of *C. parapsilosis* is urgently needed^12^. Horizontal transmission, lack of primary colonization, rapid growth in parenteral nutrition and its common occurrence among the neonates are additional major features of *C. parapsilosis*^13,14^. Despite of the availability of applicable molecular tools for gene disruption^15,16^, only a limited number of studies are available about the virulence attributes of this species^17–19^. To date, extensive mutant libraries are now available for *C. albicans*, for the purpose of studying transcriptional regulators, although only one is accessible in *C. parapsilosis*. A *C. parapsilosis* deletion mutant library including more than a hundred regulatory ORFs, with approximately 37% of mutant strains shown to effect *C. parapsilosis* virulence either directly or indirectly was previously generated; however the authors only further characterized regulators that impacted biofilm formation^15^.

In this study, we aimed to investigate virulence regulatory mechanisms in *C. parapsilosis* during host-pathogen interaction. We leveraged changes in whole transcriptomes of *C. parapsilosis* upon early engagement with host effector cells to identify potential fungal regulatory factors for subsequent gene disruption and characterization. Following the generation of a targeted small mutant set based on the transcriptomic data, we aimed to characterize the mutant strains under various conditions that are thought to simulate pathogenesis with the purpose of searching for mutants with multiple defects. Among the identified regulators, we highlight novel transcriptional regulators that influence pathogenicity determining mechanisms in distinct ways in this species.

## Results

### Identification of *C. parapsilosis* virulence regulatory genes

In order to identify virulence regulatory factors, THP-1 monocytes were infected with *C. parapsilosis* cells using a multiplicity of infection (MOI) of 5. Following co-incubation, host cells were removed after 1 and 6 hours post-infection and fungal RNA was isolated for whole transcriptome analysis using Illumina-based sequencing (see Materials and Methods). In addition, we also considered yeast cells incubated in the same medium but in the absence of THP-1 cells as a control. We used a state- of- the- art pipeline (see Materials and Methods) to analyze the RNA sequencing reads. Our results show clear changes in expression upon incubation with THP-1 cells and during the monitored time course, with an increase in expression of fungal genes during host interaction, suggesting their involvement in virulence (see Materials and Methods, Supplementary Fig. S2 and Supplementary Table S1). A total fold change greater than 4 in gene expression (log2fold change greater than 2) was used to select genes for further analyses (Supplementary Table S1). The set of up-regulated genes includes several uncharacterized ORFs with hypothetical regulatory functions ranging from transcriptional factors to protein kinases, according to orthology-based functional assignment. Furthermore, the set of up-regulated genes included an additional ORF, *CPAR2_501400*, for which orthology-based functional assignment suggests a role in cell wall beta 1,6-glucan assembly. Based on their expression profiles and their putative biochemical activities, we aimed to generate a set of deletion mutant strains (Table 1).

**Table 1:** Targets of the *C. parapsilosis* deletion mutant library.

	Fungal genes with elevated expression after	
	1 hour	6 hour
Transcriptional factor	*CPAR2_200040*	
with Zn finger motif	*CPAR2_602820*	
	*CPAR2_400270*	
	*CPAR2_108410*	
Transcriptional factor	*CPAR2_401150*	
	*CPAR2_104420*	
	*CPAR2_302400*	
	*CPAR2_200390*	*CPAR2_200390*
	*CPAR2_202040*	*CPAR2_202040*
	*CPAR2_100540*	*CPAR2_100540*
	*CPAR2_602430*	*CPAR2_602430*
Kinase	*CPAR2_300080*	
	*CPAR2_209520*	
	*CPAR2_502720*	*CPAR2_502720*
	*CPAR2_303700*	*CPAR2_303700*
	*CPAR2_108840*	*CPAR2_108840*
	*CPAR2_304080*	*CPAR2_304080*
		*CPAR2_500180*
Other	*CPAR2_501400*	*CPAR2_501400*

### Preparation of deletion mutant strains

In order to study the role of the up-regulated hypothetical regulators in *C. parapsilosis* virulence, we established a deletion mutant collection of the identified ORFs. ORF deletion was performed using the fusion PCR method^20^, as adapted to *C. parapsilosis* by Holland *et al*.^15^. Due to its high specificity and speed, this method allowed us to generate a set of deletion mutant strains of the selected genes, each represented by two parallel homozygous deletion mutant strains. Following confirmation of the null mutants, strains were prepared for phenotypic assays.

### Systematic screen of the generated mutant strains

#### Viability testing and response to stressors

**Figure 1:**
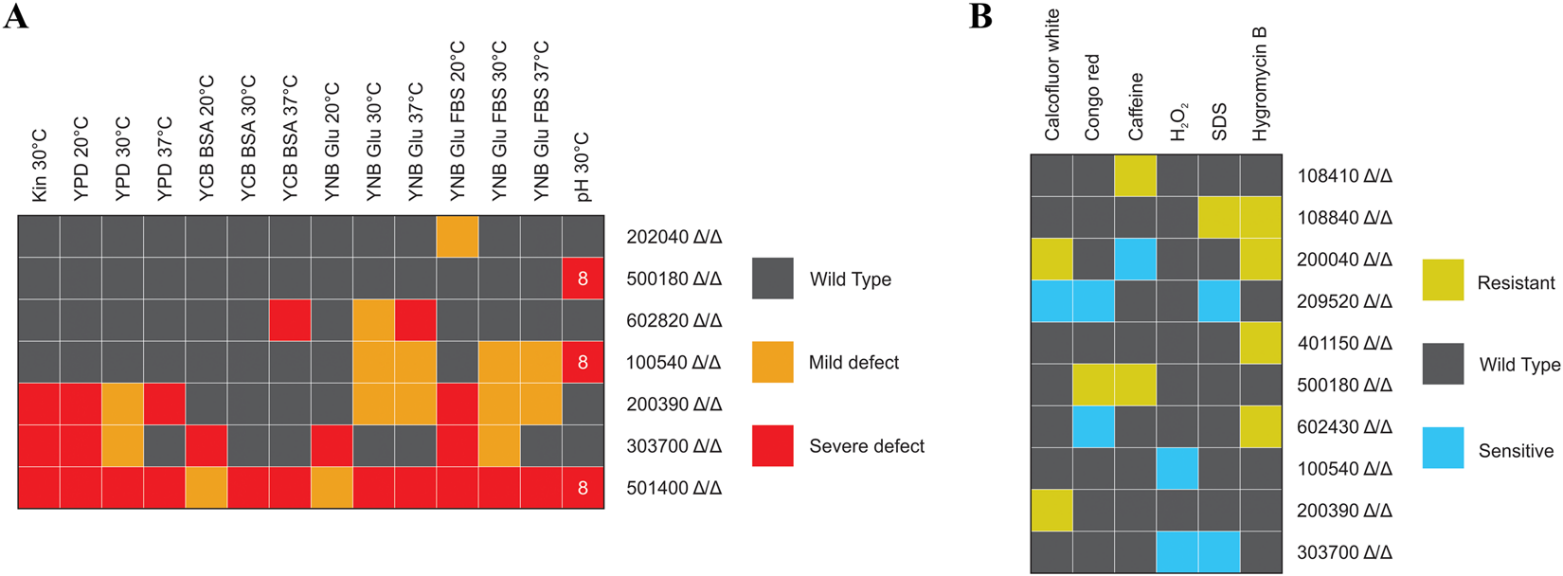
Screening of the *C. parapsilosis* mutant strains. Summary table of the mutant phenotypes identified through viability testing on different solid media (**A**) and screening of stress responses in stressor supplemented liquid solutions (**B**). Mutant strains with altered phenotypes are shown only. Screening conditions are detailed in the Materials and Methods section and in Supplementary Materials and Methods. Defective growth, sensitive and resistant phenotypes were determined relative to the CLIB 214 wild-type strain.

During invasion, fungal cells need to adapt to restrictive environmental conditions in order to survive and disseminate throughout a host. Such conditions include the presence of alternative nitrogen and carbon sources, elevated temperature, and a shift from neutral pH to slightly basic or acidic^21–23^. Pathogenic species also developed strategies to degrade antimicrobial components present in the host serum^19,24^. Therefore, the deletion mutant strains were first analyzed in terms of fitness and viability under various conditions. Among the tested mutant strains, three showed a general growth defect on YPD complex media, indicating an adverse effect on fungal viability (Fig. 1/A). Furthermore, five showed reduced growth on minimal media (YNB + glucose), three on complex media set to pH 8, three in the presence of bovine serum albumin (BSA), and five on fetal bovine serum (FBS) supplemented plates, suggesting a potential defect in adaptation to alkaline conditions, in alternative energy source utilization and serum protein degradation (Fig. 1/A). The response of each deletion mutant strain was also examined in the setting of various restrictive environmental conditions^25^. Survival of each mutant strain was evaluated in the presence of different stressors in liquid media. During fungal infection, phagocytic cells first distinguish cell wall surface components of the invaders via pattern recognition receptors, thus cell wall assembly significantly influences virulence^26^. To assess alterations in cell wall homeostasis calcofluor white, congo red and caffeine were used^27,28^. To identify mutants with potential glycosylation defects, Hygromycin B was also applied^29^. According to our results, eight strains showed altered response to the aforementioned stressors (Fig. 1/B). To evaluate the responses of the mutants to oxidative damage, we used H_2_O_2_ as stressor, to which two strains were susceptible (Fig. 1/B). In order to examine cell membrane integrity the membrane stressor SDS was used. During the analyses three mutant strains showed altered susceptibility to the membrane disturbing agent (Fig. 1/B). Altogether, these data suggest that 13 of the 19 examined genes may be involved in viability and stress tolerance regulation.

#### Morphology change

**Figure 2:**
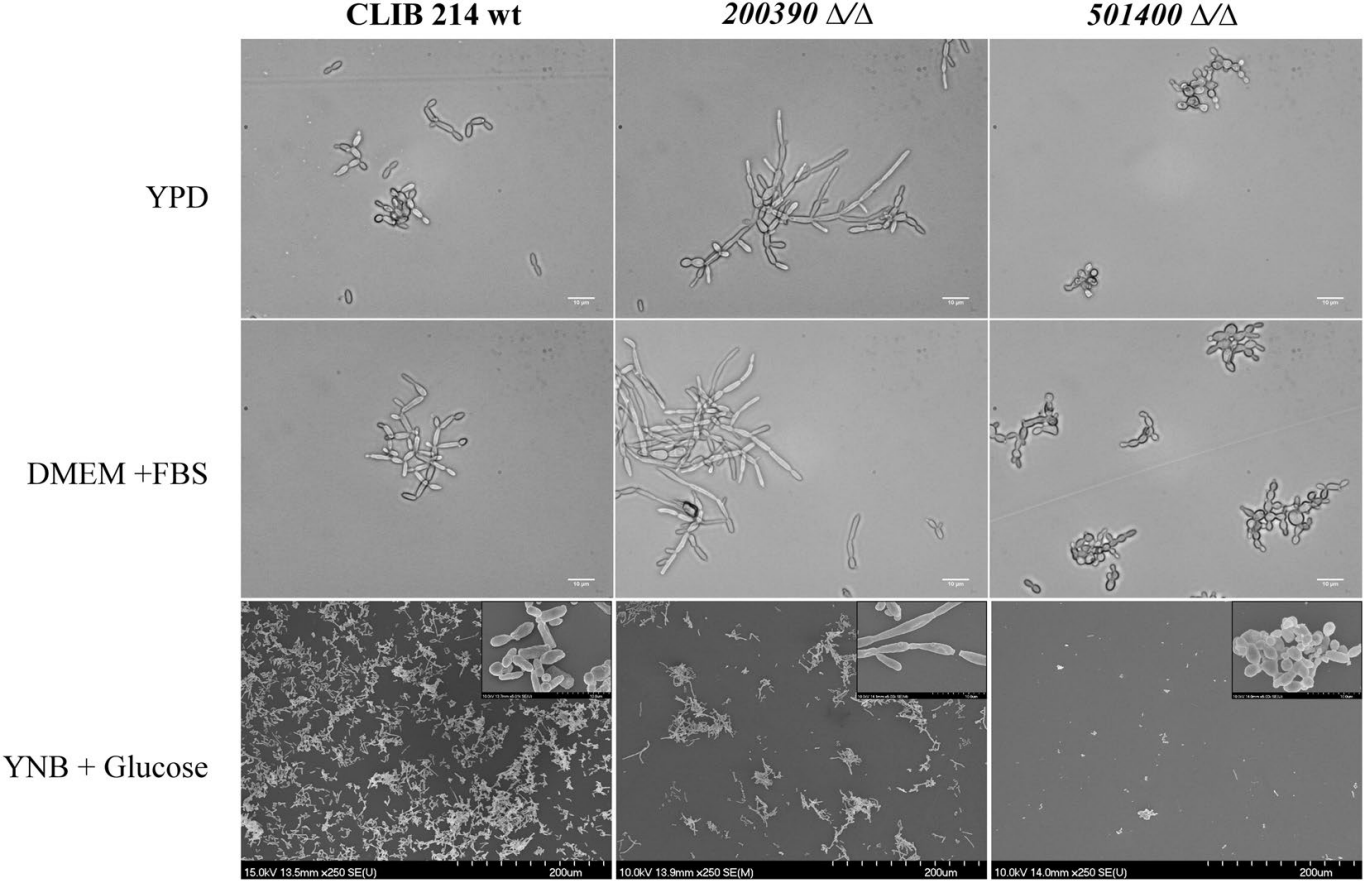
Pseudohyphal growth of the *C. parapsilosis* mutant strains. DIC and SEM images of pseudohyphae produced by the wild-type (CLIB 214) *CPAR2_200390Δ/Δ* and *CPAR2_501400Δ/Δ* strains 48 hours after cultivation in YPD, FBS supplemented DMEM and YNB + glucose liquid medium. Scale bars: 10 µm and 200 µm.

Although *C. parapsilosis* is unable to form true hyphae, the morphogenic shift to pseudohyphal forms has been associated with virulence^13^. When examining the ability of the mutants to form pseudohyphae, we found that *CPAR2_200390Δ/Δ* and *CPAR2_501400Δ/Δ* strains showed a remarkably different phenotype compared to the wild type. Yeast cells of *CPAR2_200390Δ/Δ* rapidly transitioned into extremely long and aggregating pseudohyphae, while *CPAR2_501400Δ/Δ* cells remained phenotypically locked in a yeast form (Fig. 2). Thus, these data suggest that *CPAR2_200390* and *CPAR2_501400* may be involved in the regulation and maintenance of morphology.

#### Biofilm formation and adhesive properties

**Figure 3:**
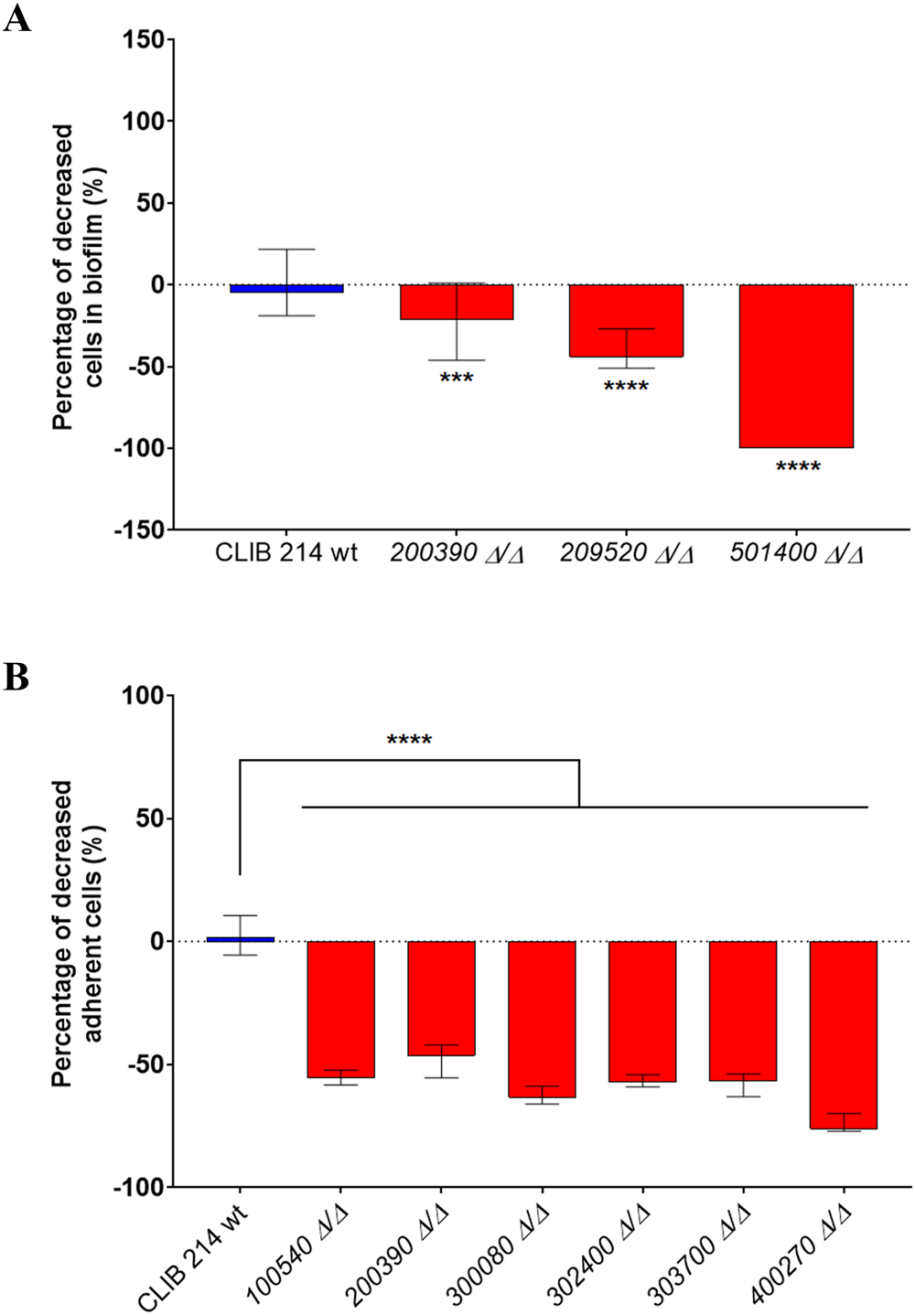
Biofilm formation and substrate adherence of the *C. parapsilosis* mutant strains. (**A**) - Biofilm formation in microtiter plates was determined using FDA assay. For mutants N ≥ 16 and for CLIB wt N = 196, from at least 2 independent experiments per strain were used. Obtained data were analyzed by Kruskal-Wallis and Dunn’s multiple comparisons tests (***p ≤ 0.001; ****p ≤ 0.0001). **(B) -** Adherence to polystyrene plastic as a substrate in microtiter plates was also measured. For adherence assays N ≥ 12 for all mutant strains and N = 36 for the wild type strain were used from at least 2 independent experiments per strain and analyzed by Kruskal-Wallis and Dunn’s multiple comparisons test (****p ≤ 0.0001).

*C. parapsilosis* effectively forms biofilms on intravenous catheters, prostheses, and other indwelling medical devices^13^. In order to examine the biofilm forming abilities of each deletion mutant strain we applied the FDA metabolic assay (Supplementary Fig. S3 and Supplementary Table S2). Our results demonstrated that three strains showed a different biofilm profile when compared to the CLIB 214 wild-type strain (Fig. 3/A). Strains *CPAR2_200390Δ/Δ, CPAR2_209520Δ/Δ* and *CPAR2_501400Δ/Δ* displayed a lower capacity for biofilm formation, suggesting that the corresponding genes influence biofilm formation in *C. parapsilosis*.

Adhesion to various biotic and abiotic surfaces is a critical step for biofilm formation. While several key regulators of adhesion have been identified in *C. albicans*, only a few have been described in *C. parapsilosis*^17^. In order to search for such regulators, we tested adhesion to polystyrene plastic, the substrate used for the aforementioned biofilm formation assay (Supplementary Fig. S4 and Supplementary Table S2.). According to our results, six of the examined strains (*CPAR2_100540Δ/Δ, CPAR2_200390Δ/Δ, CPAR2_300080Δ/Δ, CPAR2_302400Δ/Δ, CPAR2_303700Δ/Δ* and *CPAR2_400270Δ/Δ*) showed decreased ability for adhesion (Fig. 3/B). As *CPAR2_501400Δ/Δ* cells were unable to significantly adhere to the polystyrene substrate, the results obtained with this strain were excluded from the final analyses.

### Determining gene function - selected mutant strains

Following the systematic screening of our mutant strains, three interesting ORFs with ≥6 altered phenotypes were selected for further in-depth analysis as potential regulators of distinct virulence-affecting mechanisms during host-pathogen interactions. These were nutrient acquisition (*CPAR2_100540*), morphology switch, cell wall reassembly (*CPAR2_200390*), and viability (*CPAR2_303700*).

#### Nutrient acquisition by *CPAR2_100540*

**Figure 4:**
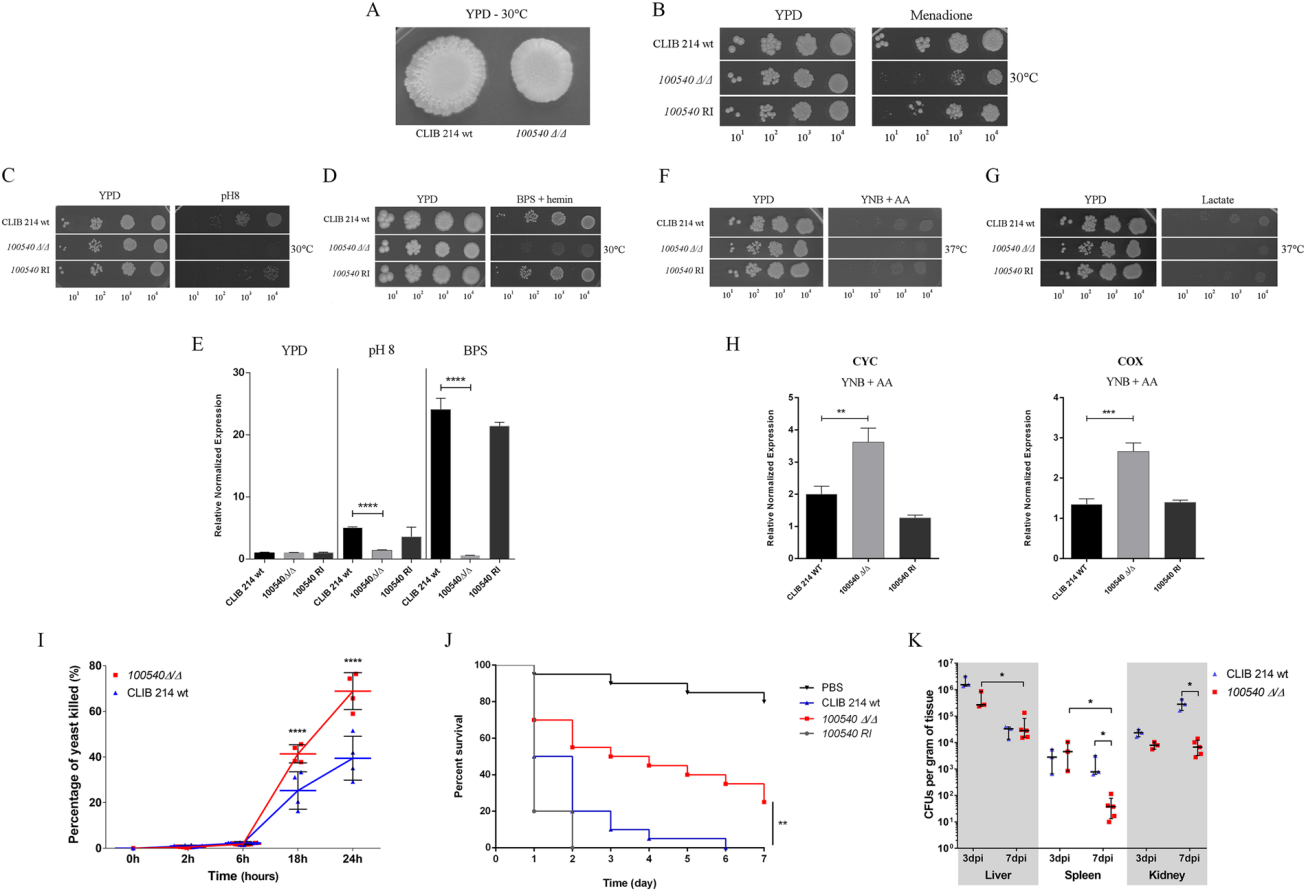
Phenotypic traits of the *CPAR2_100540Δ/Δ* strain. (**A**) Colony morphology on YPD solid medium after 2 days of incubation at 30 °C. Growth of wild type (CLIB 214), *CPAR2_100540Δ/Δ* and *CPAR2_100540* reintegrant (RI) strains on (**B**) 0.015 mM menadione containing solid medium after 3 days, (**C**) on pH 8 medium after 2 days, (**D**) on 500 mM BPS + 2 μM hemin supplemented media following 6 days, (**F**) on YNB + amino acid (AA) supplemented and (**G**) 2% lactate containing plates after 3 days of incubation, at the presented temperatures. Following growth assays, gene expression levels of the (**E**) *C. albicans FRP1* ortholog, *CPAR2_402880* was determined after cultivation of the three strains in both inductive (pH 8, BPS supplemented YPD) and non-inductive (YPD) conditions for 12 hours at 37 °C. (**H**) Relative normalized expression of *CYC* and *COX* gene orthologs (*CPAR2_407500* and *CPAR2_207710*) was also examined following cultivation in YPD and amino acid supplemented (AA) YNB medium at 37 °C. Statistical significance was determined by unpaired two-tailed *t*-tests (**p ≤ 0.01; ***p ≤ 0.001, (****p ≤ 0.0001). (**I**) Interaction between J774.1 macrophage-like cells and yeast mutants was followed for 24 h, with determinations made at 2, 6, 18, and 24 h. For 2 h N ≥ 5, 6 h N = 8, and for 18 h and 24 h N = 4, from 2 independent experiments. Virulence properties of the mutant strains was also investigated *in vivo*, examining (**J**) *G. mellonella* larvae survival and (**K**) organ colonization of BALB/c mice after infection. Statistical significance was determined by two-way ANOVA and Dunnett’s multiple comparisons tests in case of J774.1, unpaired Mann-Whitney test in case of BALB/c mice infection and Mantel-Cox (Log-rank) test after *G. mellonella* infection (*p ≤ 0.05; ****p ≤ 0.0001).

During the general characterization of the deletion mutant strains, the *CPAR2_100540Δ/Δ* strain produced smooth colony morphology (Fig. 4/A), showed a mild growth defect on minimal media (Fig. 1/A) and appeared to be defective in terms of adhesion (Fig. 3/B) when compared to the wild type. *CPAR2_100540Δ/Δ* cells were also more susceptible to oxidative stress (Fig. 4/B) and alkaline environmental conditions (Fig. 4/C) than the wild type. While the inability to grow on pH 8 suggests a failure in trace element (e.g. ferric or ferrous iron) acquisition, oxidative stress susceptibility may indicate defects in respiration. The orthologous gene of *CPAR2_100540* in *C. albicans* (*HAP5*, 68.0% amino acid sequence identity) is known to play a role in both processes.

Iron acquisition: *C. albicans* Hap5, an indispensable subunit of the CBF (CCAAT-binding factor) complex, is required for the expression of the essential iron reductase *FRP1* under iron-limited conditions^30,31^. Loss of *CaHAP5* results in low *FRP1* expression levels when cells are grown in inducing media with either pH 8 or an iron chelator^30^. Furthermore, iron chelators inhibit the growth of *Cahap5Δ/hap5Δ* cells^30^. Similarly, *C. parapsilosis CPAR2_100540Δ/Δ* has decreased growth at pH 8 (Fig. 4/C) and on iron chelator (BPS)-supplemented media that also contained hemin (low-iron source) (Fig. 4/D). Gene expression analysis suggests that iron acquisition by *C. parapsilosis* is also dependent on a ferric reductase (*CPAR2_402880*) similar to *C. albicans FRP1. CPAR2_402880* expression in *C. parapsilosis* is also induced under both iron-limited conditions in the wild type strain (Fig. 4/E). In contrast, *CPAR2_402880* expression levels remain relatively low in the *CPAR2_100540Δ/Δ* mutant strain, thus further supporting a role of *CPAR2_100540* in *C. parapsilosis* iron acquisition (Fig. 4/E).

Alternative carbon source utilization: Carbon utilization via respiration is tightly related to available iron^32^, therefore the phenotypic traits observed in this section might be also linked to the altered iron homeostasis. Previous reports suggested that *HAP5* is involved in alternative carbon source utilization in *S. cerevisiae* and *C. albicans*^33,34^. Alternative energy source metabolism is usually associated with altered regulation of respiratory chain element encoding genes such as cytochrome c (*CYC*) subunits and/or cytochrome c oxidases (*COX*)^33,34^. Thus, growth deficiencies on alternative carbon sources are often related to defects in the respiratory chain^35^. Susceptibility of *CPAR2_100540Δ/Δ* cells to oxidative stressors further suggests a respiratory chain defect. When studying the role of *CPAR2_100540* in unfermentable carbon source metabolism, *CPAR2_100540Δ/Δ* cells showed decreased growth on both amino acids (Fig. 4/F) and lactate-supplemented media (Fig. 4/G). Furthermore, gene expression analysis results suggest that the observed phenotype may be due to the misregulation of ORFs equivalent to *CYC1* (*CPAR2_407500*) and *COX4* (*CPAR2_207710*) of *C. albicans* (Fig. 4/H). Therefore, the obtained data led us to the conclusion that *CPAR2_100540* is also involved in alternative carbon source utilization via regulating elements of the respiratory chain.

Contribution of *CPAR2_100540* to virulence: Competition for the available iron sources and the ability to utilize alternative energy sources in the host are considered important virulence traits of pathogenic fungi^36^. To investigate the effects of the *CPAR2_100540* ORF on virulence, we used both *in vitro* and *in vivo* infection models. Killing assays performed with J774.1 macrophage-like cells indicated that more of *CPAR2_100540Δ/Δ* yeast cells were killed when compared to the wild-type strain both at 18 h and 24 h of the interaction (Fig. 4/I). Due to structural and functional similarities between *G. mellonella* and the mammalian innate immune system, this non-vertebrate model is frequently used as an alternative for virulence studies of *Candida* species^37^. Following inoculation with *C. parapsilosis* strains, survival of the individual larvae was monitored for 7 days. The survival results showed that the loss of *CPAR2_100540* ORF resulted in significantly decreased virulence compared to the wild type strain (Fig. 4/J). We confirmed the attenuation of *CPAR2_100540Δ/Δ* in a murine infection model. Infection with these cells resulted in significantly lower fungal burdens in the spleen and kidney 7 days after challenge, when compared to mice infected with the wild type strain (Fig. 4/K). These data confirm the role of *CPAR2_100540* in *C. parapsilosis* virulence, possibly via the regulation of iron utilization and alternative carbon source acquisition.

#### Morphology switch and cell wall assembly regulation by *CPAR2_200390*

**Figure 5:**
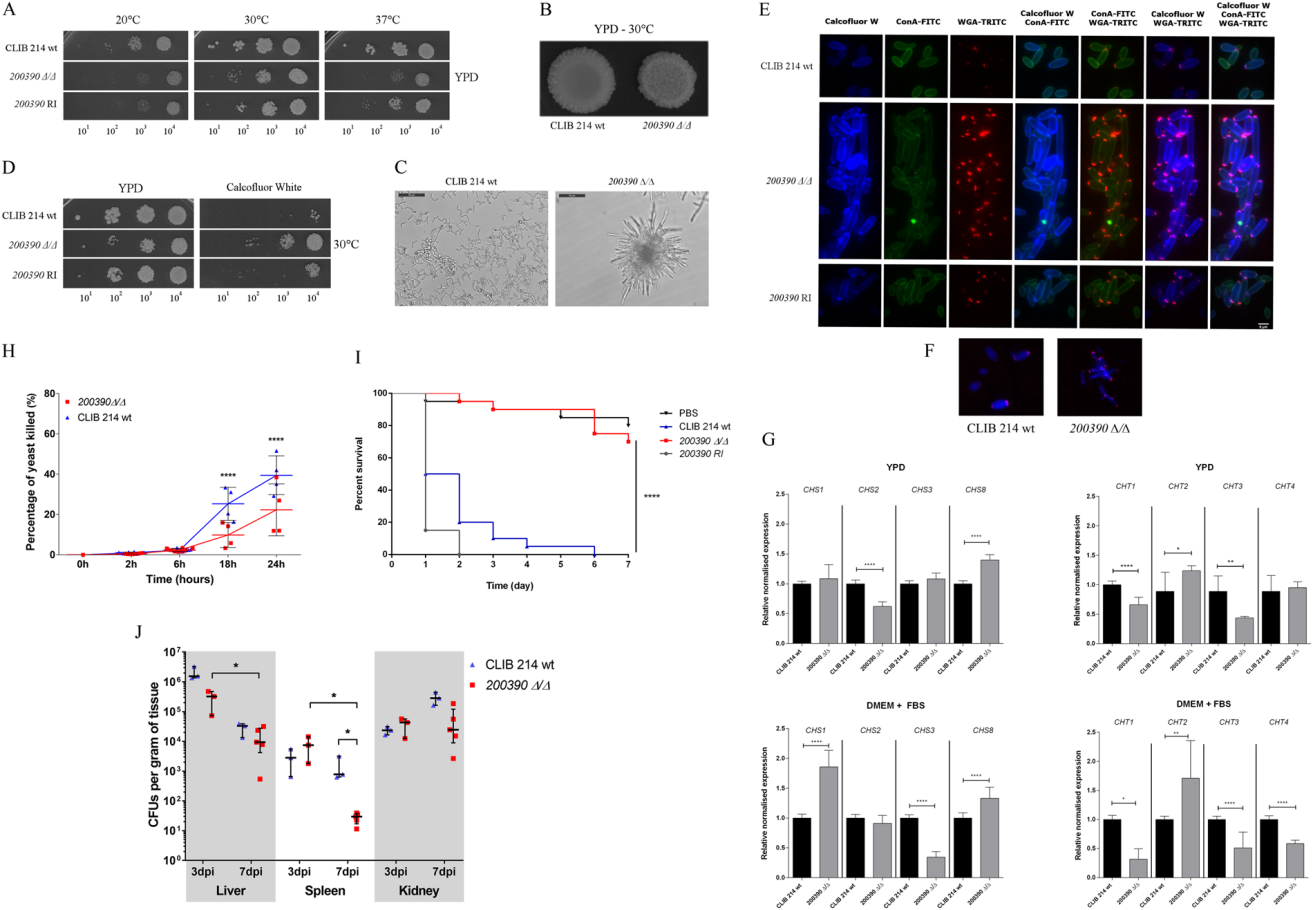
Characteristics of the *CPAR2_200390Δ/Δ* strain. (**A**) Growth of wild-type (CLIB 214), *CPAR2_200390Δ/Δ* and *CPAR2_200390* reintegrant (RI) strains after 2 days on YPD solid media at selected temperatures. (**B**) Colony morphology on YPD plate following 2 days of incubation at 30 °C. **(C)** Pseudohyphae formation in 10% FBS supplemented DMEM medium after 24 hours of incubation at 37 °C. (**D**) Growth on 60 μg/ml calcofluor white supplemented YPD medium at 30 °C. (**E**) Fluorescence microscopic images of calcofluor white, ConA (Concanavalin A) and WGA (wheat germ agglutinin) stained wild-type, *CPAR2_200390Δ/Δ* and *CPAR2_200390* RI cells. (**F**) Localization of chitin oligomer accumulations in the wild-type and mutant cell wall. (**G**) Relative normalized expression of *C. albicans* chitinase and chitin synthase orthologs (*CPAR2_805640* as *CHS1; CPAR2_701490* as *CHS2; CPAR2_801800* as *CHS3; CPAR2_502940* as *CHS8, CPAR2_800050* as *CHT1; CPAR2_ 502140* as *CHT2, CPAR2_ 200660* as *CHT3* and *CPAR2_211950* as *CHT4)* after cultivation in YPD and 10% FBS supplemented DMEM medium. Statistical significance was determined by unpaired two-tailed *t*-tests (*p ≤ 0.05; **p ≤ 0.01; ****p ≤ 0.0001). Virulence attributes of the *CPAR2_200390Δ/Δ* strain following (**H**) an *in vitro* killing assay with J774.1 macrophage-like cells and after (**I**) *G. mellonella* larvae and (**J**) BALB/c mice infection. Statistical significance was determined by two-way ANOVA and Dunnett’s multiple comparisons tests in case of J774.1, and unpaired Mann-Whitney test in case of BALB/c mice infection. Mantel-Cox (Log-rank) tests were applied in the case of *G. mellonella* infection (*p ≤ 0.05; ****p ≤ 0.0001).

Morphology transition: Our general characterization of the *CPAR2_200390Δ/Δ* strain revealed a retardation in growth (Fig. 5/A) along with an irregular colony morphology (Fig. 5/B) and hyper-filamentation (Fig. 5/C), a phenotype set similar to that observed in *C. albicans* following the removal of its orthologous gene, *SPT3* (81.8% amino acid sequence identity)*. C. albicans SPT3* is a negative regulator of filamentous growth^38^. The obtained phenotypic traits suggested functional homology between *SPT3* and the *CPAR2_200390* ORF, supporting the inclusion of *CPAR2_200390* as a regulator of morphogenesis in *C. parapsilosis*. Interestingly, during the characterization of the *CPAR2_200390Δ/Δ* mutant strain we also observed low adhesive and biofilm forming capability (Fig. 3/A-B) along with resistance to the cell wall stressor calcofluor white (Fig. 5/D). The latter observation led us to the assumption that *CPAR2_200390* in *C. parapsilosis* might also be involved in the maintenance of cell wall homeostasis.

Cell wall rearrangement: In order to further examine the cell wall stress resistant phenotype of *CPAR2_200390Δ/Δ* cells, different fluorescent dyes were used to investigate cell wall composition with calcofluor white binding to chitin, Concanavalin A (ConA) to alpha mannan, and Wheat Germ Agglutinin (WGA) to chitin oligomer compounds. Fluorescent labeling revealed that the *CPAR2_200390Δ/Δ* strain displayed elevated chitin and chitin oligomer content in comparison with the wild type (Fig. 5/E). In addition to the accumulation of chitin oligomers around apical bud scars, the oligomers were also present along the longitudinal line of the mutant cells (Fig. 5/F) suggesting altered chitin homeostasis in *CPAR2_200390Δ/Δ*. To support this hypothesis, we examined the expression level of four chitinase and four chitin synthase encoding genes that were potentially involved in the dysregulation. Examined chitinase encoding ORFs included those equivalent to *C. albicans CHS1 (CPAR2_805640), CHS2 (CPAR2_701490), CHS3 (CPAR2_801800)* and *CHS8 (CPAR2_502940)*, while chitinase encoding genes were *CPAR2_800050 (CHT1), CPAR2_ 502140 (CHT2), CPAR2_ 200660 (CHT3)* and *CPAR2_211950 (CHT4)*^39–42^. Real-time PCR analyses revealed altered chitinase and chitin synthase expression profiles for the mutant strain in both YPD complex and 10% FBS supplemented DMEM- medium when compared to the wild type (Fig. 5/G). These data suggest that the *CPAR2_200390* ORF is also involved in chitin homeostasis regulation.

Role of *CPAR2_200390* in virulence: During fungal infection, cell wall assembly has a major impact on recognition by immune cells, while a morphology switch is associated with host cell and tissue disruption^36^. In killing assays performed with J774.1 macrophage-like cells, the *CPAR2_200390Δ/Δ* strain had increased survival compared to wild-type cells both at 18 h and 24 h of interaction (Fig. 5/H). Interestingly however, *G. mellonella* infection studies suggested that *CPAR2_200390Δ/Δ* cells are avirulent *in vivo*, as survival rates were similar to that of PBS infected larvae (Fig. 5/I). Furthermore, following infection of BALB/c mice, the mutant cells had reduced CFUs present in the spleen 7 days after infection, compared to wild type-infected animals (Fig. 5/J). Hence, *CPAR2_200390* plays a role in morphology transition and cell wall homeostasis maintenance, and also contributes to virulence.

#### Fitness regulation by *CPAR2_303700*

**Figure 6:**
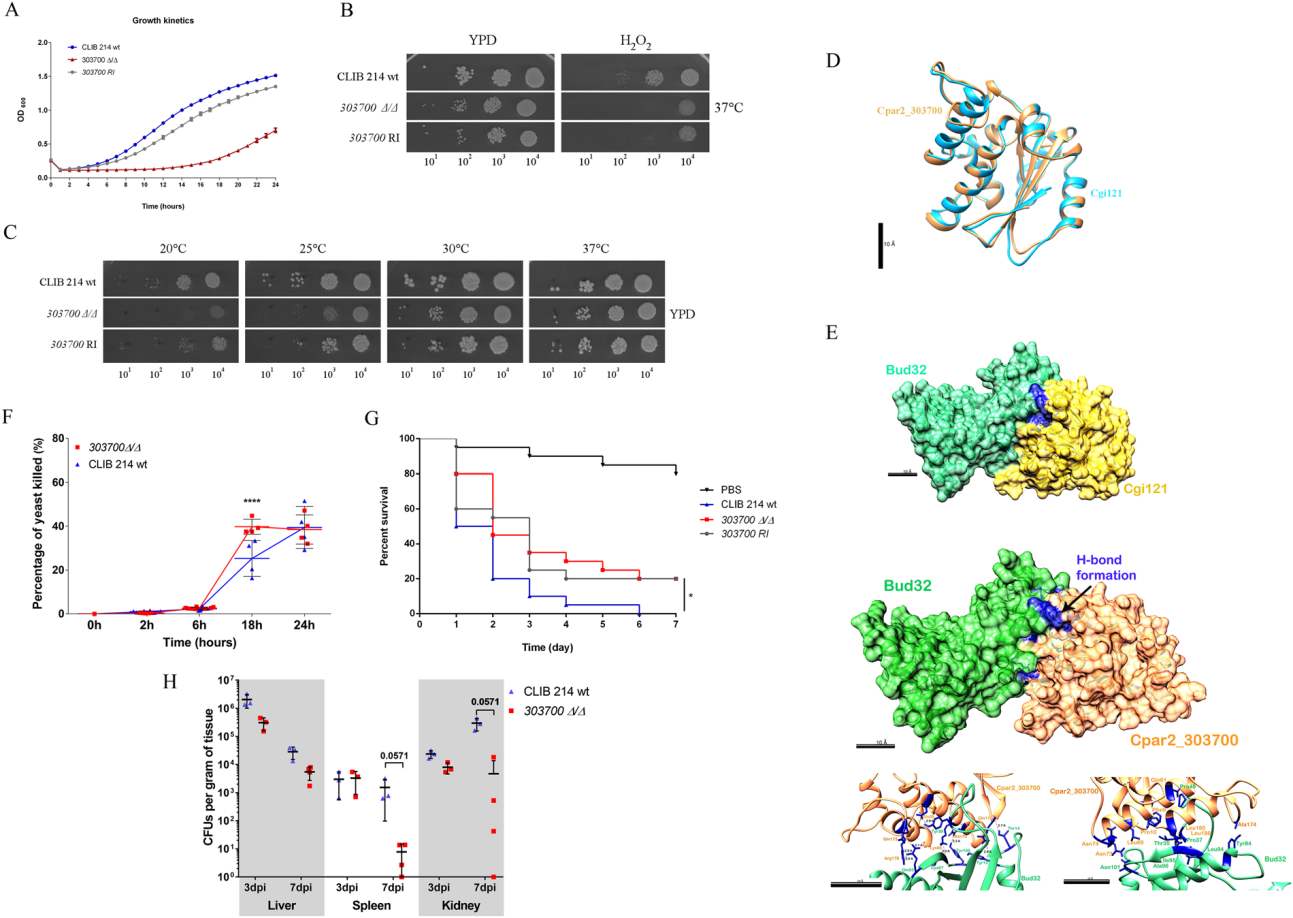
Phenotypic features of the *CPAR2_303700Δ/Δ* strain. (**A**) Growth kinetics of wild-type (CLIB 214), *CPAR2_303700Δ/Δ* and *CPAR2_303700* reintegrant (RI) strains in YPD liquid medium, at 30 °C. (**B**) Growth on 10 mM H_2_O_2_ supplemented YPD medium, and (**C**) on YPD solid medium at different temperatures. (**D**) *In silico* 3D structure comparison of the hypothetical Cpar2_303700 and the previously described *S. cerevisiae* Cgi121 proteins. Scale bar: 10 ångström (Å). (**E**) 3D structure of the complex formed by Cpar2_303700 and Bud32 and its comparison to the Cgi121-Bud32 complex. Predicted H-bonds (bottom left) and the surrounding hydrophobic residues (bottom right) maintain the stability of the Cpar2_303700 – Bud32 complex. Moderately attenuated virulence of *CPAR2_303700Δ/Δ* (**F**) *in vitro* after J774.1 macrophage infection, following (**G**) *G. mellonella* larvae inoculation and (**H**) BALB/c mice infection. Statistical significance was determined by two-way ANOVA and Dunnett’s multiple comparisons tests in case of J774.1, unpaired Mann-Whitney tests in case of BALB/c mice infection and Mantel-Cox (Log-rank) test after *G. mellonella* infection: *p ≤ 0.05; ****p ≤ 0.0001.

Viability regulation: Notable features of the *CPAR2_303700Δ/Δ* mutant strain included a growth defective phenotype (Fig. 6/A), low adhesive properties (Fig. 3/B), and susceptibility to the oxidative stressor H_2_O_2_ (Fig. 6/B) and to decreased temperature (Fig. 6/C). The closest characterized orthologue of *CPAR2_303700* is *S. cerevisiae CGI121*, encoding a subunit of the KEOPS/EKC complex^43–45^. According to preliminary searches, this factor is conserved across the entire *Candida* clade and Cpar2_303700 also contains conserved domains of the *CGI121* superfamily (NCBI conserved domain search). According to the performed *in silico* data analyses (Supplementary Materials and Methods), the two proteins share similar secondary and tertiary structures (Fig. 6/D, Supplementary Table S3) and Cpar2_303700 is likely to form a stable conformation with the closest interacting partner of Cgi121 in the *S. cerevisiae* KEOPS/EKC complex, Bud32 (Fig. 6/E, Supplementary Table S4.), suggesting that Cpar2_303700 and Cgi121 may also share similar properties. Structural similarity and the predicted interaction between Cpar2_303700 and Bud32 was supported by measurable values such as low average RMSD values used for distance comparison (0.61 ± 0.04 Å in case of structure comparison and 0.4 ± 0.2 Å in the predicted complex) and negative overall energy levels (Supplementary Table S4). *In silico* 7 H-bonds were observed between the residues of Cpar2_303700 and Bud32 that were stabilized by several hydrophobic interactions, further supporting a stable interaction (Fig. 6/E). These data also suggest that Cpar2_303700 might be a potential member of the *C. parapsilosis* KEOPS/EKC complex.

*CPAR2_303700* and virulence: In *A. nidulans*, members of the KEOPS/EKC complex are associated with metabolic processes that are known to influence the virulence properties of *C. albicans*. These mechanisms include the regulation of *TUP1*’s function as a key yeast to filamentous growth switch regulating transcriptional factor as well as the complex’s involvement in amino acid and carbon source acquisition^46–49^. Sensitivity to oxidative stress and to temperature changes is also known to impact the pathogenicity of *Candida* species^36^. Our results indicate that the *CPAR2_303700Δ/Δ* mutant strain was more susceptible to killing by J774.1 macrophage-like cells, in particular at 18 h of interaction when compared to the wild type strain (Fig. 6/F). When examining survival rates of *G. mellonella* larvae we found that the mutant cells were less virulent compared to the wild type (Fig. 6/G). The intermediate phenotype of the 30700 revertant (RI) strain could be due to the gene dosage effect or to local regulatory effects present at the target locus used for reintegration. BALB/c infection results further suggested reduced virulence of the mutant strain as a reduction was also observable in the number of CFUs recovered from the spleen (p = 0.0571) and the kidney (p = 0.0571) 7 days after the infection (Fig. 6/H). These data suggest that the *CPAR2_303700* ORF could also contribute to *C. parapsilosis* virulence.

## Discussion

To date, several kinase inhibitors are in use against certain diseases, mainly acting as ATP competitive inhibitors, and lead transcriptional factor inhibitory drugs have already passed preclinical studies, either interfering with the dimerization, co-factor or DNA binding properties of their targets^50,51^. Thus, transcriptional regulators are attractive ‘ligands’ for novel drug design in various diseases. Due to the complexity of pathogenic mechanisms, multifunctional regulatory factors such as transcriptional factors and kinases are as important as virulence factors themselves in determining the outcomes of host-pathogen interactions. Therefore, targeting the virulence regulatory machinery of pathogenic fungal species has been proposed as an innovative method for effectively combating fungi while concomitantly avoiding the toxicity and resistance mechanisms of the currently available antifungal compounds^2^.

In this study, we used a pre-selective approach for target selection in order to identify regulators potentially contributing to the virulence of an emerging human fungal pathogen, *C. parapsilosis* in various routes. To identify such regulatory genes, whole RNA sequencing was performed shortly following host-pathogen interaction. Among the ORFs upregulated after infection, transcriptional factor- and kinase-encoding genes were selected for further examination and a targeted deletion mutant collection was constructed. These deletion mutant strains were tested under various conditions designed to recapitulate environments the fungus would encounter under certain host conditions or that would likely influence *C. parapsilosis* pathogenicity in an indirect manner. As a result we found that 84% of the characterized mutant strains had a phenotype different from wild-type (Supplementary Fig. S5, Supplementary Table S5). Furthermore, 32% of the tested mutant strains showed multiple phenotypic defects suggesting that the examined regulators have pleiotropic effects (Supplementary Fig. S5). Out of the characterized ORFs, three with potentially distinct regulatory roles in virulence were further examined: *CPAR2_100540*, *CPAR2_200390* and *CPAR2_303700*.

During infection, pathogens continually compete with host cells for available iron sources. Although iron compounds are usually carried by high affinity proteins in the host, limiting access to these trace elements, pathogenic fungi have developed various strategies in order to acquire these vital compounds^52^. In addition, carbon utilization via respiration is tightly linked to available iron - due to the presence of iron containing enzymes –, that further highlights the role of this trace element in viability^32^. Depending on the site of invasion, invading fungi face a limited access to glucose and often only alternative carbon sources are available for utilization. Under such circumstances, alternative carbon and nitrogen source metabolic pathways are activated, which usually require additional energy and, thus, alter the function of the mitochondrial respiratory chain^49,53^. In *C. albicans* both iron acquisition and alternative carbon source metabolism are influenced by the transcriptional factor, Hap5, a subunit of the conserved CBF complex. Hap5 controls iron homeostasis by regulating the ferric reductase *FRP1*, and also influences carbon source utilization via regulating respiratory chain elements cytochrome c and cytochrome c oxidases^30,34^. According to our results, the identified *CPAR2_100540* ORF, an orthologous gene of *HAP5*, plays a similar role in both processes in *C. parapsilosis*. Moreover, results of the performed virulence studies indicate that the identified regulator further contributes to the virulence of this species in both *in vitro* and *in vivo* infection models. It is noteworthy to mention that *S. cerevisiae HAP5*, is also involved in alternative carbon source utilization, although, while *CYC* and *COX* gene expression was downregulated in the *S. cerevisiae hap5Δ/hap5Δ* strain, upregulation was observed in the respective mutant of *C. albicans*, which underscores the variability in gene regulation between different yeast species and supports further investigation of these processes in other fungi^33,34^. In the present work, our functional studies show that *CPAR2_100540* is comparable to that of *C. albicans HAP5*; thus, this identified regulator is likely required for *C. parapsilosis* virulence via the regulation of nutrient acquisition and alternative carbon source utilization.

Yeast to pseudohyphal growth transition promotes *C. parapsilosis* invasion through several mechanisms, including host cell and tissue disruption, tissue penetration and biofilm formation^13^. In this study, loss of the *CPAR2_200390* ORF resulted in a phenotypic feature set previously observed after the removal of *SPT3* in *C. albicans*. Spt3, a subunit of the evolutionally conserved SAGA complex, is known to play a key role in the morphological switching of both *S. cerevisiae* and *C. albicans*^38^. Although while *SPT3* deletion resulted in a yeast-locked phenotype in *S. cerevisiae*, hyper-filamentous growth was observed in *C. albicans*, suggesting opposite regulatory functions in the two species^38^. The obtained phenotype set of the *CPAR2_200390Δ/Δ* strain indicated that *CPAR2_200390* function is equivalent to that of *C. albicans SPT3*. Interestingly, our further analyses revealed that *CPAR2_200390* also influences the chitin homeostasis of the cell wall, adhesive properties and biofilm formation, suggesting a pleiotropic effect on *C. parapsilosis* virulence. These features have not been previously associated with *SPT3*. Furthermore, our virulence studies underscored the influence of this regulator on *C. parapsilosis* pathogenicity, although the outcomes were inconsistent, as the *CPAR2_200390Δ/Δ* strain was significantly attenuated *in vivo*, however less efficiently killed by murine macrophages *in vitro*, when compared to the wild type. These results suggest that at the cellular level, clearance of *CPAR2_200390 Δ/Δ* is less dependent on macrophages due to weak or no preference (decreased phagocytic activity, data not shown), although could be the result of an alternative host response. In total, these data confirm that *CPAR2_200390* regulates virulence in *C. parapsilosis* by mechanisms that affect mainly morphogenesis and cell wall assembly.

Several conditions, such as temperature and oxidative stress, are known to influence fungal viability, which in turn indirectly affects virulence. Removal of *CPAR2_303700* resulted in various defects, mainly including a general growth defective phenotype coupled with sensitivity to low temperatures, suggesting the regulator’s involvement in fungal fitness. The closest ortholog to the identified *C. parapsilosis* ORF is *S. cerevisiae*’s *CGI121*, and there is no ortholog yet characterized among other *Candida* species. As part of the highly conserved KEOPS/EKC complex, Cgi121 is involved in functions such as transcription co-activation, tRNA modification, and telomere maintenance, although not required for survival in *S. cerevisiae*^43–45^. Although the performed *in silico* data analyses do not confirm functional similarity between Cgi121 and Cpar2_303700, they suggest that Cpar2_303700 is a protein structurally similar to Cgi121. Thus Cpar2_303700 may also be a subunit of the evolutionarily conserved KEOPS/EKC complex in *C. parapsilosis*.

Interestingly, disruption of 3 of the 5 KEOPS/EKC subunits in *S. cerevisiae* resulted in a serious growth defective phenotype and temperature sensitivity, features that are especially common among telomere defective strains^43^. Mutants defective in telomere maintenance are also hypothesized to be more susceptible to oxidative stress, although this phenotype was not reported in with *CGI121*. In addition, removal of *CGI121* rescued growth defective phenotypes of telomere defective mutant strains, which led to the conclusion that Cgi121 promotes telomere uncapping^43^. The evolutionally conserved hypothetical function and phenotypical features of *CPAR2_303700Δ/Δ* and *Cgi121Δ/Δ* strains suggest that there might be opposite regulatory mechanisms between *C. parapsilosis* and *S. cerevisiae*, similarly to what was observed in case of *HAP5* and *SPT3*^34,38^, although this hypothesis needs to be supported by further experiments and analyses. Although the exact function of *CPAR2_303700* is not yet determined, according to our results, the identified ORF nevertheless contributes to virulence regulation, possibly via an indirect manner.

Taken together, in this study we identified several interesting *C. parapsilosis* mutant strains with relevant virulence-determining features and a relatively high yield of mutants with multiple defects that indicate the respective regulatory gene’s function in virulence. Out of the characterized genes, we describe three transcription regulators that influence *C. parapsilosis* pathogenicity in distinct ways and thus contribute to our better understanding of virulence regulation in this species. These include Cpar2_100540, a transcriptional factor regulating nutrient acquisition, Cpar2_200390 involved in morphology switch and cell wall assembly, and Cpar2_303700, a protein kinase regulating fungal viability. Although more in-depth studies of the identified regulatory processes in *C. parapsilosis* are required, these data further support the idea that the origin of virulence in pathogenic species can be due to alterations in signaling and regulatory networks.

## Materials and Methods

### THP-1 cell line infection with *C. parapsilosis* cells

THP-1 monocytic cells (Sigma-Aldrich, 100 μl of 5 × 10^6^/ml) were seeded onto 96 well plates in 10% FBS (EuroClone) supplemented Dulbecco’s Modified Eagle Medium (DMEM, Lonza) and incubated overnight at 37 °C, with 5% CO_2_. Infection of monocytes with *C. parapsilosis* CDC 317 cells was performed in 1:5 ratio. Cells were co-incubated at 37 °C with 5% CO_2_ and collected by centrifugation (1000 rpm, 5 min) at 1 or 6 h post-infection. Cells were washed once with ice-cold PBS and pellet was suspended in RNase-free distilled water. For fungal RNA isolation, monocytes were lysed through 27 G needles and RNA extraction was performed with the RNeasy Plant Mini Plus Kit (Qiagen) according to manufacturer’s instructions.

### RNA sequencing and data analysis

RNA sequencing libraries were prepared according to protocols provided by Illumina. Sequencing was carried out in an Illumina HiSeq2000 sequencer with a depth of >20 million reads per sample at the CRG genomics facility. We sequenced paired-end 50bp-long reads RNAseq data for the above-mentioned time-points. The reference genomes for the reference strain CDC317 and the human hg19 were obtained from the *Candida* Genome Database (CGD)^54^ and UCSC (UCSC Genome Browser)^55^, respectively. Gene annotations were obtained from the gencode project, version 18 and from CGD. Read mapping and alignment were carried out using tophat2 v2.0.9^56^ with default sensitivity and specificity conditions, based on the bowtie2 2.1.0 short read mapper^57^. Transcript abundance estimation was obtained via FluxCapacitor v1.5.2 with automatic annotation mapping. Gene normalization for thresholding was carried out via RPKM (Reads Per Kilobase of transcript per Million mapped reads) obtained from Flux Capacitor output. A minimum threshold of RPKM ≥10 was used to eliminate low expression transcripts and limit noise. Differential expression analysis was carried out via the DESeq2 v1.10.1 Differential Expression analysis package in R.

### Preparation of *C. parapsilosis* mutant strains

Strains used in this study are listed in Supplementary Table S6. Two of the 19 deletion mutants were also part of the Holland deletion collection and analysis (*CPAR2_202040* and *CPAR2_100540*). Homozygous mutant strains were generated according to methods described by Holland *et al*. using the *C. parapsilosis* CLIB 214 double auxotrophic strain (CPL2H1)^15^. Primers used for gene deletion and null mutant confirmation are presented in Supplementary Dataset 1. In each case, two homozygous deletion mutant strains were selected for phenotypic analyses.

For the mutant strains *CPAR2_100540Δ/Δ, CPAR2_200390Δ/Δ* and *CPAR2_303700Δ/Δ*, one copy of the respective allele was introduced into the *RP10* locus for complementation (see Supplementary Materials and Methods)^58^.

### Strain cultivation

Strains were grown in YPD liquid medium (1%D-glucose-1%peptone-0.5%yeast-extract) supplemented with 1% Penicillin-Streptomycin with shaking (200 rpm) at 30 °C overnight, unless stated otherwise. Detailed information about the characterization process is included in Supplementary Materials and Methods.

### Response to oxidative, cell wall and other stressors

Cells from overnight cultures were collected, washed 3× with PBS and diluted with fresh YPD to the desired concentration, and 95 μl of 5 × 10^4^cells/ml suspension was plated into each well of 96-well plates. In liquid media: 5 µl of the stress inducing reagents was added to each well from a serial of two-fold dilutions. Detailed information about the applied concentrations of stress inducing reagents is freely accessible and listed in Supplementary Materials and Methods file. Survival rates were normalized to the values of cells grown in YPD media only and compared to the wild-type strain. Survival curves were determined after end-point measurements performed at OD_600_ following 18 h of incubation at 30 °C. At least two individual experiments were performed using at least three parallels.

For validation of *CPAR2_100540Δ/Δ* and *CPAR2_200390Δ/Δ* altered phenotypes, additional stressor containing YPD solid media were also used (Supplementary Materials and Methods). At least two individual experiments were performed to confirm obtained phenotypes.

### Pseudohypha formation

For the experiments, 100 µl of 10^7^cells/ml suspension of each strain was added into 24-well cell culture plates, containing 1 ml YPD or DMEM (Gibco) supplemented with 10% heat-inactivated FBS and 1% Penicillin-Streptomycin. Strains were grown at 37 °C and the morphologies were documented at 24 and 48 h of incubation using Leica light microscope (DMI4000B, Leica Microsystems) and HITACHI S-4700 cold field emission scanning electron microscope (see Supplementary Materials and Methods for sample preparation). Three individual experiments were performed to confirm abnormal phenotypes.

### Cell wall composition assay (microscopy)

To examine cell wall composition of *C. parapsilosis* mutant cells, ConA-FITC (Sigma-Aldrich), calcofluor white (Sigma-Aldrich) and WGA-TRITC (Sigma-Aldrich) were used. A detailed protocol of staining procedure is available in Supplementary Materials and Methods. Stained strains were imaged with a Zeiss Observer Z1 fluorescence microscope. At least three individual experiments were performed to confirm alterations in cell wall assembly.

### Adhesion and biofilm assay

Cultures were grown overnight (12–16 h) at 30 °C with shaking in YNB medium. Cells were washed with PBS and adjusted to 10^5^cells/ml. In a microtiter plate, 100 µL of adjusted cultures were aliquoted per well and statically incubated at 37 °C for 90 min for adhesion to occur. Each well was washed once with 100 µL of PBS, and metabolic activity assay (FDA) was performed to assess adhesion capacity. For adhesion assays, N = 36 for wild-type and N ≥ 12 for mutant strains was used from at least 2 independent experiments. In case of biofilm formation, following adhesion of seeded cells, each well was washed once with 100 µL of PBS, and 100 µL of fresh YNB media was added per well. Plates were incubated at 37 °C with a wet paper towel for 48 h for biofilms to form. After biofilm formation, each well was washed with 100 µL of PBS and biofilm levels were assessed using a metabolic activity assay (FDA). For biofilm assays, at least 2 independent experiments were performed for each mutant strain.

### Fluorescein diacetate (FDA) Assay

To each tested well, 100 µL of FDA solution (40 µg/mL, Sigma-Aldrich) was added. Plates were incubated at 37 °C for 1 h, protected from light, and supernatants were transferred to a new fluorescence-compatible plate and read at 485/535 nm (ex/em) in a Victor plate reader.

### Gene expression analyses

For total RNA isolation, the Ambion, Ribopure^TM^-Yeast RNA isolation kit (Invitrogen) was applied. Prior to examining expression levels in BPS, pH8, amino acid supplemented YNB and FBS-containing DMEM medium, 50 µl of 2 × 10^7^cells from overnight cultures were transferred into the respective media and incubated overnight at 37 °C. For gene expression analysis during biofilm formation, cells were grown in 1 ml of 0.5% glucose-supplemented YNB solution for 48 h.

For cDNA synthesis, the RevertAid First Strand cDNA Synthesis Kit (Thermo Scientific) was applied according to the manufacturer’s guide. For expression analyses 100ng cDNA were used. PCR conditions were as follows: 95 °C for 3 minutes, followed by 50 cycles of 95 °C for 10 s, and 60 °C for 30 s. Primers used for gene expression studies are listed in Supplementary Dataset 1. *TUB4* housekeeping gene was used as an internal control. Data were normalized to wild-type gene expression levels. Three individual experiments were performed to confirm alterations in gene expression.

### Interaction between *C. parapsilosis* and J774.1 macrophage-like cells

Killing assays were performed according to a standardized protocol with minor modifications^59^. Briefly, 5 × 10^4^ J774.1 macrophages per well (American Type Culture Collection) were infected with *C. parapsilosis* cells in a 5:1 ratio of yeast:macrophage-like cells. Control wells contained yeast cells only. Interactions were left for 0, 2, 6, 18, and 24 h at 37 °C, with 10% CO_2_. Contents were collected, washed, and passed through a 26 G needle. Suspensions were plated on Sabouraud dextrose agar plates and incubated at 30 °C for 24–48 h. Killing efficiency was calculated as described previously^59^. Graph values were normalized to the number of viable fungal cells in untreated wells at 24 h (100%). At 2 h N ≥ 5, 6 h N = 8, and at 18 h and 24 h N = 4 from 2 independent experiments were used.

### *Galleria mellonella* infection assay

Infection was achieved with 10 µl of 5 × 10^8^ *C. parapsilosis* cells/ml inoculated in the last pro-leg and 20 caterpillars (N = 20) were used per strain. Groups of PBS sham-infected and uninfected (witness group) larvae were also used. The *Galleria* were maintained at 30 °C and survival was monitored daily.

### *In vivo* murine infection model

Female Balb/c mice (Jackson Laboratories, 6–8 weeks of age, NCI) were injected intraperitoneally with 10^7^cells (N ≥ 6 per yeast strain). After 3 days of infection, 3 mice per condition were sacrificed and the remaining mice were sacrificed at day 7 post-infection. Liver, kidney, and spleen were collected by necropsy, weighed, homogenized, and plated on Sabouraud dextrose agar plates. Colony forming units (CFUs) were counted after 24 h incubation at 30 °C, to assess infection levels.

### Ethics Statement

Animal experiments were performed according to the guide published by the Institute of Laboratory Animal Resources of the National Research Council. Animal care for this study was approved by the Animal Welfare and Research Ethics Committee at the Albert Einstein College of Medicine (Animal Use Protocol #20170503).

### Statistical analyses

Unpaired, two-tailed *t*-tests were applied for gene expression analysis, Kruskal-Wallis test with Dunn’s multiple comparisons test was performed for FDA assays, 2-way ANOVA and Dunnett’s test for multiple comparisons was applied for J774.1, and non-parametric Mann-Whitney tests were performed on BALB/c mice data assessment, and Mantel-Cox (Log-rank) tests were used for survival data evaluation. After unpaired, two-tailed *t*-tests, the mean ± SEM values, whereas in case of non-parametric tests, medians and the interquartile range are represented on the graphs. Statistical significance was determined by GraphPad Prism v5.0 or v6.0software. Significant differences were considered at *P*-values of ≤ 0.05(*p ≤ 0.05; **p ≤ 0.01; ***p ≤ 0.001; ****p ≤ 0.0001).

## Electronic supplementary material

Supplementary Information

Dataset 1
