# Supplementary material for: Investigation of Candida parapsilosis virulence regulatory factors during host-pathogen interaction
Source: Sci Rep. 2018 Jan 22;8:1346. doi: 10.1038/s41598-018-19453-4 (PMC5777994; doi:10.1038/s41598-018-19453-4)
Supplement: Supplementary file 1 — Supplementary Information [file 41598_2018_19453_MOESM1_ESM.pdf]

Investigation of *Candida parapsilosis* virulence regulatory factors during host-pathogen interaction

Renáta Tóth[1†], Vitor Cabral[2†] , Ernst Thuer[3,4], Flóra Bohner[1], Tibor Németh[1], Csaba Papp[1], Leonardo Nimrichter[6], Gergő Molnár[1], Csaba Vágvölgyi[1], Toni Gabaldón[3,4,5], Joshua D. Nosanchuk[2] and Attila Gácser[1*]

1. Department of Microbiology, University of Szeged, Szeged, Hungary

2. Departments of Medicine and Microbiology and Immunology, Albert Einstein College of Medicine, New York, NY, USA

3. Centre for Genomic Regulation (CRG), Barcelona Institute of Science and Technology, Barcelona, Spain

4. Universitat Pompeu Fabra (UPF), Barcelona, Spain.

5. Institució Catalana de Recerca i Estudis Avançats (ICREA), Barcelona, Spain.

6. Laboratório de Glicobiologia de Eucariotos, Instituto de Microbiologia Professor Paulo de Góes, Universidade Federal do Rio de Janeiro, Rio de Janeiro, Brazil

Supplementary Materials and Methods

***In silico* data analysis of *S. cerevisiae* Cgi121 and *C. parapsilosis* Cpar2_303700.**

In order to compare Cgi121 and Cpar2_303700 protein structure, the 3D structure of Cpar2_303700 was predicted by the Modbase server and database. Fortunately, the crystal structure of *S. cerevisiae* Cgi121 protein was published by Zhang et al. (2015). For comparing the predicted tertiary structure of Cpar2_303700 and Cgi121 we used the Chimera software. Visualized alignments of the two 3D structures suggested a high structural similarity between Cgi121 and Cpar2_303700 that was further supported by the given RMSD value of 0.61 ± 0.04 Å (usually <2 Å for distance comparison of the respective alpha carbonic atoms of similar proteins 10/D). Negligible alignment differences were due to variations in the number of secondary motifs. Predicted structure of Cpar2_303700 contained 1 beta sheet and 4 loop regions less than Cgi121 (Supplementary Table 3). *In silico* analysis on the HADDOCK server was performed to predict the probability of docking between Cpar2_303700 and Bud32. Among the 200 conformations predicted by HADDOCK, the majority of interactions (96.5%) fall into one cluster with the average values of characteristics shown in Supplementary Table 4. Protein-protein interactions are considered stable if the distance between the interacting residues is quite low (<3.5 Å) and the formed stabile conformation usually requires relatively low energy levels (≤0 kcal/mol).

Preparation of reconstituted strains for *CPAR2_100540Δ/Δ*, *CPAR2_200390Δ/Δ* and *CPAR2_303700Δ/Δ*

Reintegration of each of the genes was achieved using the Gateways system adapted to *C. parapsilosis* (see Supplementary Information 2.) Briefly, the amplified target gene with its native promoter and terminator region (primers REFw and RERev, S5 Table) was cloned into the plasmid vector pDONR220, via the BP cloning reaction, and later transferred into the *CpRP10* integration site and *NAT* dominant selection marker containing modified destination vector via LR cloning. The resulting expression vectors were then used for transformation. Restored expression levels were verified by real time PCR (RT Fw and RT rev primers, Supplementary Table 7.).

Applied conditions for deletion mutant strain characterization.

Before testing, cells were washed 3x with phosphate buffered saline (PBS) and diluted to the desired concentration. During the general characterization of each mutant strain, 5µl of $2x10^6$cells/ml, $2x10^5$cells/ml, $2x10^4$cells/ml and $2x10^3$cells/ml suspension was plated onto different solid media listed in Supplementary Information 2. The viability of the strains was examined after 48h of incubation at 20, 30, and 37°C.

Growth kinetics were also examined at 30°C, after 24h of incubation in YPD liquid medium with measurements performed hourly at OD_{600}. At least two individual experiments were performed per condition to confirm phenotypes.

General viability testing of all mutants

Applied medium	Temperature
YPD	20 °C, 30 °C, 37 °C
2% BSA+1.17% YCB	20 °C, 30 °C, 37 °C
1% glucose + 0.67% YNB w/o amino acids	20 °C, 30 °C, 37 °C
1% glucose + 0.67% YNB + 10% FBS	20 °C, 30 °C, 37 °C
YPD - pH4, 5,6,7,8 - set with McIlvaine buffer	30 °C, (37 °C)

Conditions used for characterizing the *CPAR2_100540Δ/Δ* strain on solid medium:

Iron starvation test - *CPAR2_100540Δ/Δ:*

- 2μM hemin (Sigma – Aldrich, Darmstadt, Germany) combined with 500mM BPS (4,7-diphenyl-1,10-phenanthrolinedisulfonic acid, Sigma – Aldrich, Darmstadt, Germany) supplemented solid YPD medium; 150mM BPS supplemented solid YPD medium

Alternative carbon source utilization test - *CPAR2_100540Δ/Δ:*

- amino acid supplemented 0.67% YNB solid medium
- 2% lactate solid medium

Applied stressor concentrations for survival tests in liquid medium:

Congo red: 100 μg/ml; 50 μg/ml; 25 μg/ml; 12.5 μg/ml; 6.25 μg/ml; 3.125 μg/ml

Calcofluor white: 100 μg/ml; 50 μg/ml; 25 μg/ml; 12.5 μg/ml; 6.25 μg/ml; 3.125 μg/ml

Caffeine: 50 mM; 25 mM; 12.5 mM; 6.25 mM; 3.125 mM; 1.562 mM

H_2O_2: 7.5 mM; 3.75 mM; 1.875 mM; 0.937 mM; 0.468 mM; 0.234 mM

SDS:.0.1 %; 0.05 %; 0.025 %; 0.0125 %; 0.006 %; 0,003 %

Hygromycin B: 15.6 μg/ml; 7.8 μg/ml; 3.9 μg/ml; 1.95 μg/ml; 0.975 μg/ml; 0.487 μg/ml

Applied stressor concentrations for survival tests on solid medium:

Congo red: 60 μg/ml; 50 μg/ml; 40 μg/ml; 30 μg/ml; 20 μg/ml; 10 μg/ml

Calcofluor white: 60 μg/ml; 50 μg/ml; 40 μg/ml; 30 μg/ml; 20 μg/ml; 10 μg/ml

Caffeine: 10mM, 5mM and 2.5mM

SDS: 0.25%, 0.125% and 0.06%

Hygromycin B: 6 μg/ml, 3 μg/ml, and 1 μg/ml

H_2O_2: 10mM and 5mM

As an alternative oxdative stressor - $CdSO_4$: 0.005mM; Menadione: 0.015mM

Sample preparation of Scanning electron microscopy

For the experiment, 10^7/ml cells in 0.67% YNB supplemented with 0.5% glucose liquid medium were plated onto plastic coverslips (Sarstedt, Nümbrecht, Germany) and incubated at 37°C for 48 hours. The cells were immobilized with 2,5 % glutaraldehyde in 0.05 M cacodylate buffer (pH 7.5) for 2 hours and the fixed samples were then serially dehydrated with 50, 70, 80, 90 and 96% ethanol for 30 minutes each. Fixed cells were further treated with combined *t*-butanol – ethyl alcohol (1:2, 1:1, 2:1 proportion, 1 hour incubation for each) solution and then incubated in absolute *t*-butanol for 2 X 1 hours. Samples in absolute *t*-butanol were stored at 4°C overnight and then they were coated for 1min at 18 mA current resulting in a ~3 nm layer of Au-Pd target (60/40 ratio) by a sputter coater (Quorum Technologies, East-Sussex, UK, type: SC 7620 'Mini') prior to SEM viewing. SEM analyses were performed with a HITACHI S-4700 cold field emission scanning electron microscope.

Cell wall composition assay (microscopy)

After collecting the cells from overnight cultures, strains were suspended in 1ml of 4% paraformaldehyde and incubated for 30 minutes at room temperature, with continuous rotating. Following incubation, cells were washed four times with 1x PBS, and pellets were suspended in 0,5ml 1% BSA (Sigma – Aldrich), followed by incubation at room temperature for an additional 30 minutes with rotation. Strains were washed three times with PBS, and suspended in 200 - 400μl of the same buffer dependent upon cell concentration (approx. 10^8/ml cell concentration). Then, 100 μl of the suspension was transferred into a new Eppendorf tube, and mixed with 100 μl of freshly prepared CCW fluorescent dye mix. A 100ul CCW mix consists of 8 μl 2.5 mg/ml ConA-FITC, 1 μl 1mg/ml CW, 1 μl 1mg/ml WGA-TRITC, 90 μl 1% BSA. Cell suspensions were incubated for 30 minutes at room temperature with continuous rotation. The fluorescently labelled cells were washed three times with 1xPBS and suspended in 100μl of the same buffer and kept on ice covered until use.

Supplementary Fig. S1. Complexity of the pathogenicity system. Cartoon depicting of known virulence regulatory and virulence factors of *C. albicans* and their contribution to fungal pathogenesis.

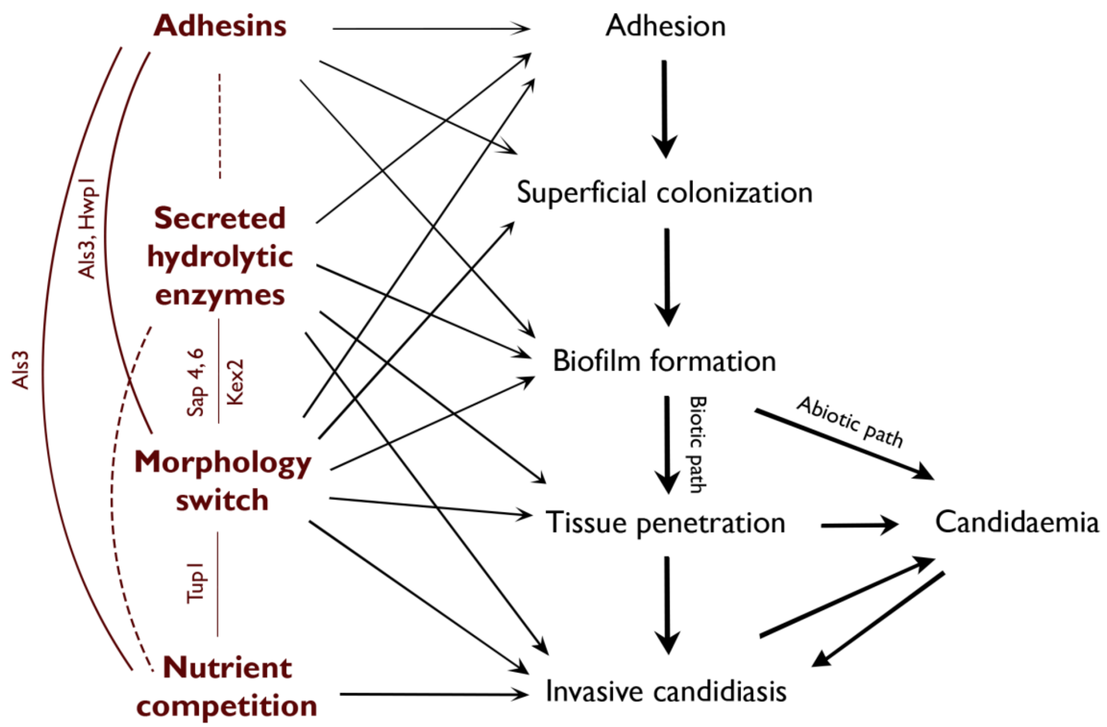

Supplementary Fig. S2. Expression of fungal genes following host – pathogen interactions. For further information, see Supplementary Table S1 and Materials and Methods section.

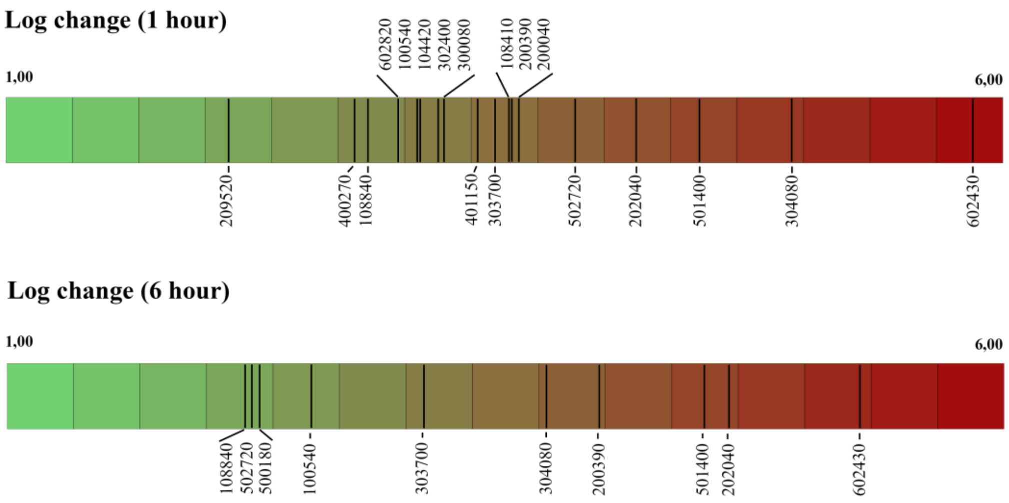

Supplementary Fig. S3. Biofilm formation by *C. parapsilosis* knockout strains. Biofilm viability determined using the FDA assay. For CLIB wt N=196, for mutants N≥16 from at least 2 independent experiments were used.

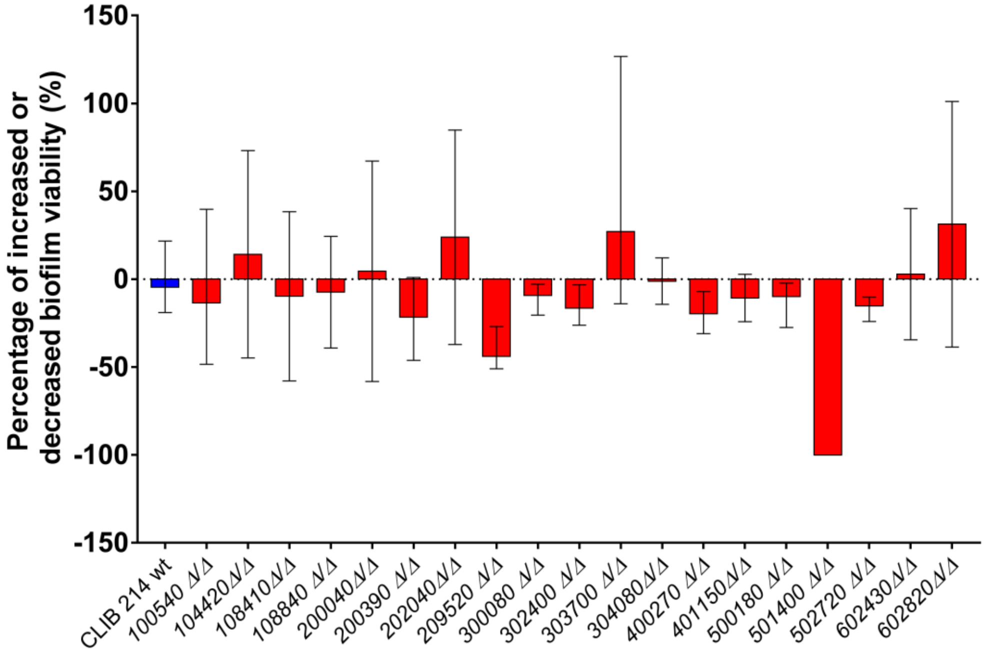

Supplementary Fig. S4. Viability of adherent cells. Adherence to polystyrene plastic of each strain was measured. For adherence assays N=36 for the wild type strain and N≥12 for all mutant strains were used from at least 2 independent experiments.

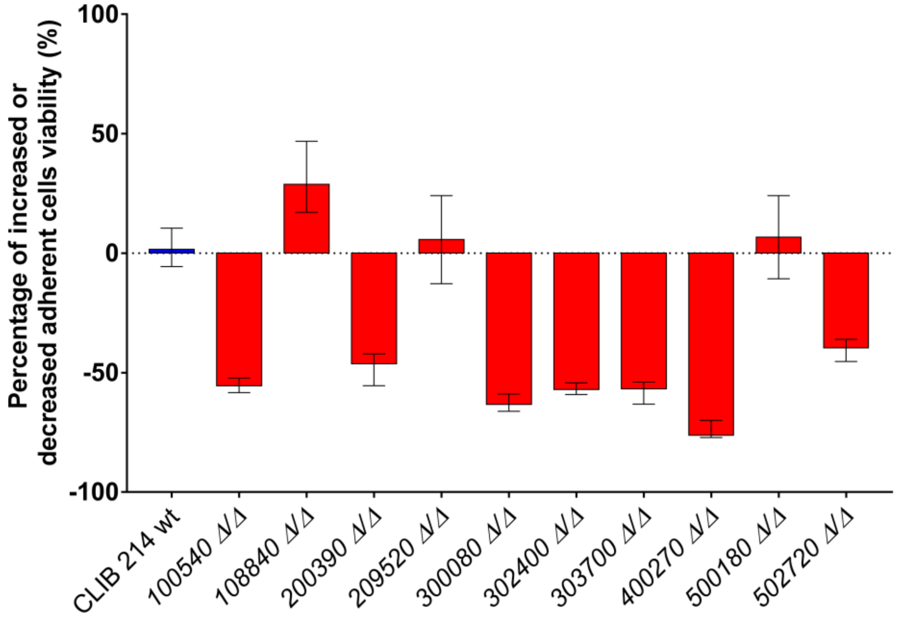

Supplementary Fig. S5. *C. parapsilosis* mutant library analysis of virulence traits. Venn diagram depicting results obtained in different virulence assays. Assays to assess virulence traits were grouped in 3 categories (Response to stressors, Growth, Adherence & Biofilm formation). Phenotypes of each strain were compared to the wild type strain and strains were grouped according to the number of different phenotypes per group of assays.

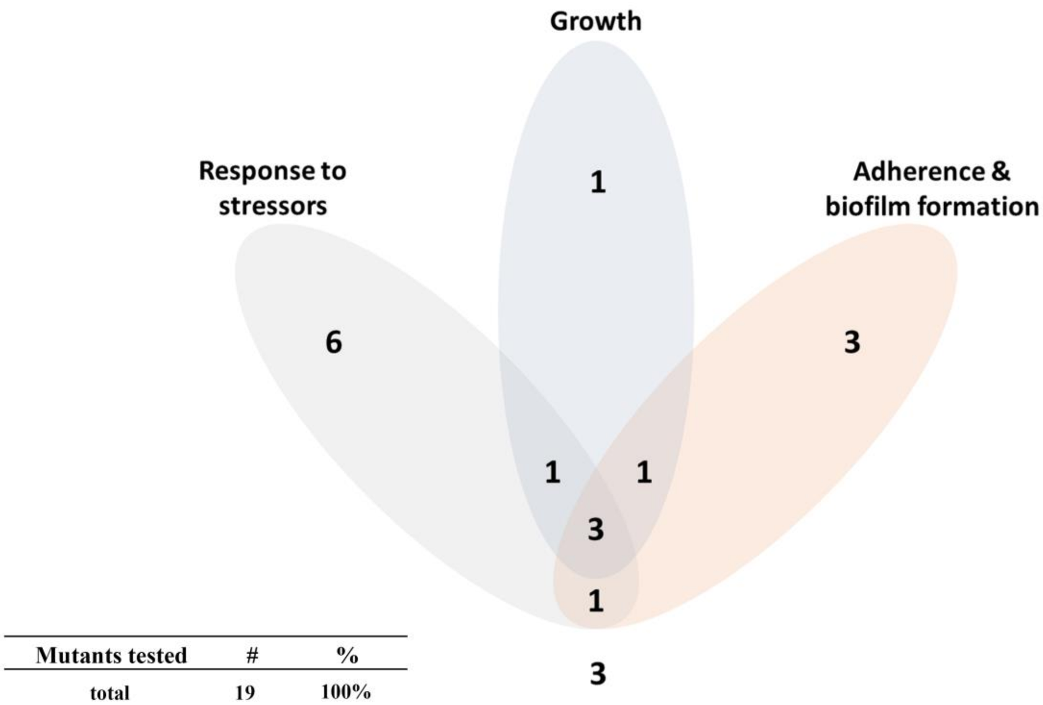

Mutants tested	#	%
total	19	100%
no phenotpye	3	16%
≥1 phenotype	16	84%
≥2 phenotype	6	32%

Supplementary table S1. RNA sequencing based Differential Expression analysis of fungal transcriptome after host-pathogen interaction.

Supplementary Table S1 shows the differentially expressed genes after host pathogen exposure between the timepoints 0h - 1h (A) and 0h – 6h (B). A threshold for the DESEq2 normalized min-count of 150 and a log2foldchange of 2.0 was applied. Results are limited to up-regulated genes. The last column describes annotated Protein Family (Pfam) domains as described at the Candida Genome Database (CGD).

Gene ID **Protein ID** **Normalized* expression count** **log change** **Pfam Domains**

Up regulated genes 1h after THP-1 - *C. parapsilosis* interaction

Gene ID	Protein ID	0 hours	1 hour	log change	Pfam Domains
CPAR2_602430	CPAG_02706	4	209	5,80	DnaJ domain [PF00226.23]; DnaJ central domain (4 repeats) [PF00684.11]
CPAR2_304080	CPAG_02165	3	90	4,94	Adenylylsulphate kinase [PF01583.12]
CPAR2_501400	CPAG_05386	3	61	4,48	Mannosyl oligosaccharide glucosidase [PF03200.8]
CPAR2_202040	CPAG_05690	7	128	4,21	Histone-like transcription factor (CBF/NF-Y) and archaeal histone [PF00808.15]
CPAR2_502720	CPAG_03844	5	73	3,84	Protein tyrosine kinase [PF07714.9]; Protein kinase domain [PF00069.17]
CPAR2_200040	CPAG_00265	77	947	3,62	LYAR-type C2HC zinc finger [PF08790.3]
CPAR2_200390	CPAG_01541	5	56	3,58	Transcription initiation factor IID, 18kD subunit [PF02269.8]
CPAR2_108410	CPAG_00937	2	20	3,57	SNF2 family N-terminal domain [PF00176.15]; Helicase conserved C-terminal domain [PF00271.23]; Zinc finger, C3HC4 type (RING finger) [PF00097.17]
CPAR2_303700	CPAG_03314	7	79	3,47	Kinase binding protein CGI-121 [PF08617.2]
CPAR2_401150	CPAG_03166	2	17	3,34	DNA mismatch repair protein, C-terminal domain [PF01119.11]; Histidine kinase-, DNA gyrase B-, and HSP90-like ATPase [PF02518.18]
CPAR2_300080	CPAG_00618	7	66	3,22	Adenylate kinase, active site lid [PF05191.6]; Adenylate kinase [PF00406.14]
CPAR2_302400	CPAG_01745	7	59	3,19	6-O-methylguanine DNA methyltransferase, DNA binding domain [PF01035.12]
CPAR2_104420	CPAG_02813	4	38	3,10	Guanine nucleotide exchange factor for Ras-like GTPases; N-terminal motif [PF00618.12]; RasGEF domain [PF00617.11]

Up regulated genes 1h after THP-1 - *C. parapsilosis* interaction

Gene ID	Protein ID	0 hours	1 hour	log change	Pfam Domains
CPAR2_100540	CPAG_02544	19	163	3,09	Core histone H2A/H2B/H3/H4 [PF00125.16]; Histone-like transcription factor (CBF/NF-Y) and archaeal histone [PF00808.15]
CPAR2_602820	CPAG_00651	43	333	2,96	Cytidine and deoxycytidylate deaminase zinc-binding region [PF00383.14]
CPAR2_108840	CPAG_00908	5	33	2,74	Protein tyrosine kinase [PF07714.9]; Protein kinase domain [PF00069.17]
CPAR2_400270	CPAG_00680	22	143	2,68	HIT zinc finger [PF04438.8]
CPAR2_209520	CPAG_01453	19	87	2,17	Protein kinase domain [PF00069.17]

Up regulation 6h after THP-1 - *C. parapsilosis* interaction

Gene ID	Protein ID	0 hours	6 hours	log change	Pfam Domains
CPAR2_602430	CPAG_02706	4	142	5,25	DnaJ domain [PF00226.23]; DnaJ central domain (4 repeats) [PF00684.11]
CPAR2_202040	CPAG_05690	7	170	4,62	Histone-like transcription factor (CBF/NF-Y) and archaeal histone [PF00808.15]
CPAR2_501400	CPAG_05386	3	61	4,50	Mannosyl oligosaccharide glucosidase [PF03200.8]
CPAR2_200390	CPAG_01541	5	72	3,94	Transcription initiation factor IID, 18kD subunit [PF02269.8]
CPAR2_304080	CPAG_02165	3	40	3,76	Adenylylsulphate kinase [PF01583.12]
CPAR2_303700	CPAG_03314	7	64	3,16	Kinase binding protein CGI-121 [PF08617.2]
CPAR2_100540	CPAG_02544	19	113	2,56	Core histone H2A/H2B/H3/H4 [PF00125.16]; Histone-like transcription factor (CBF/NF-Y) and archaeal histone [PF00808.15]
CPAR2_500180	CPAG_05269	10	51	2,36	Protein kinase domain [PF00069.17]
CPAR2_502720	CPAG_03844	5	25	2,29	Protein tyrosine kinase [PF07714.9]; Protein kinase domain [PF00069.17]
CPAR2_108840	CPAG_00908	5	24	2,25	Protein tyrosine kinase [PF07714.9]; Protein kinase domain [PF00069.17]

* DESEq normalization

Supplementary Table 2. Summary of the number of replicates and the significance obtained in Adhesion and Biofilm formation assays. (“ns" - non-significant, "nt" - not tested, statistical significance considered at *** p≤0.001; **** p≤0.0001)

	ADHESION		BIOFILM FORMATION	
**Mutant name**	N	Statistical significance	N	Statistical significance
**CLIB 214 wt**	36	-	196	-
100540 **Δ/Δ**	12	****	22	ns
104420 **Δ/Δ**	nt		20	ns
108410 **Δ/Δ**	nt		20	ns
108840 **Δ/Δ**	24	ns	44	ns
200040 **Δ/Δ**	nt		20	ns
200390 **Δ/Δ**	36	****	66	***
202040 **Δ/Δ**	nt		20	ns
209520 **Δ/Δ**	24	ns	33	****
300080 **Δ/Δ**	12	****	22	ns
302400 **Δ/Δ**	12	****	22	ns
303700 **Δ/Δ**	12	****	16	ns
304080 **Δ/Δ**	nt		22	ns
400270 **Δ/Δ**	12	****	22	ns
401150 **Δ/Δ**	nt		22	ns
500180 **Δ/Δ**	24	ns	44	ns
501400 **Δ/Δ**	24		44	****
502720 **Δ/Δ**	12	ns	22	ns
602430 **Δ/Δ**	nt		20	ns
602820 **Δ/Δ**	nt		20	ns

Supplementary Table S3. Number of secondary motiffs in *S. cerevisiae* Cgi121 and *C. parapsilosis* Cpar2_303700 proteins.

	S. cerevisiae Cgi121	*C. parapsilosis* Cpar2_303700
**α-helical**	9	9
**β-sheet**	5	4
**loop**	15	13

Supplementary Table S4. Properties of the clustered interactions between Cpar2_303700 and Bud32 proteins predicted by the HADDOCK server.

	CLUSTER 1	CLUSTER 2
**HADDOCK score**	-78.8±2.7	-65.7±3.1
**Cluster size**	193	7
**RMSD from overall lowest-energy structure**	0.4±0.2	0.5±0.0
**Van der Waals energy**	-53.8±2.8	-55.8±3.1
**Electrostatic energy**	-84.3±14.4	-86.8±15.0
**Desolvation energy**	-12.7±3.9	2.8±3.6
**Restraints violation energy**	44.8±4.79	46.0±4.66
**Buried Surface Area**	1854.8±60.3	1892±32.1
**Z-score**	-1.0	1.0

Supplementary Table S5. Summary of the multiple screens performed on the mutant collection. (+ - altered phenotype, – - no phenotype relative to the wild type strain)

**Gene name**	**Phenotype change**				
	**Response to stressors**	**Growth**		**Adhesion & Biofilm formation**	
		Growth	Morphology	Adhesion	Biofilm
CPAR2_100540	+	+	-	+	-
CPAR2_104420	-	-	-	-	-
CPAR2_108410	+	-	-	-	-
CPAR2_108840	+	-	-	-	-
CPAR2_200040	+	-	-	-	-
CPAR2_200390	+	+	+	+	+
CPAR2_202040	-	+	-	-	-
CPAR2_209520	+	-	-	-	+
CPAR2_300080	-	-	-	+	-
CPAR2_302400	-	-	-	+	-
CPAR2_303700	+	+	-	+	-
CPAR2_304080	-	-	-	-	-
CPAR2_400270	-	-	-	+	-
CPAR2_401150	+	-	-	-	-
CPAR2_500180	+	+	-	-	-
CPAR2_501400	-	+	+	-	+
CPAR2_502720	-	-	-	-	-
CPAR2_602430	+	-	-	-	-
CPAR2_602820	-	+	-	-	-

Supplementary table S6. List of strains used in this study.

Strain name	Genotype	Putative function	Reference
CLIB214			Type strain
CPL2H1 (his1- /leu2-)	*leu2::FRT/leu2::FRT, his1::FRT/his1::FRT*		L.M. Holland el al 2014
CPAR2_100540 Δ/Δ	*leu2::FRT/leu2::FRT, his1::FRT/his1::FRT, CPAR2_100540::LEU2/CPAR2_100540::HIS1*	Ortholog(s) have DNA binding, RNA polymerase II core promoter proximal region sequence-specific DNA binding, sequence-specific DNA binding transcription factor activity	This study
CPAR2_104420 Δ/Δ	*leu2::FRT/leu2::FRT, his1::FRT/his1::FRT, CPAR2_104420::LEU2/CPAR2_104420::HIS1*	Ortholog(s) have role in vesicle-mediated transport and cellular bud localization	This study
CPAR2_108410 Δ/Δ	*leu2::FRT/leu2::FRT, his1::FRT/his1::FRT, CPAR2_108410 ::LEU2/CPAR2_108410::HIS1*	Ortholog(s) have DNA-dependent ATPase activity, damaged DNA binding, ubiquitin-protein ligase activity	This study
CPAR2_108840 Δ/Δ	*leu2::FRT/leu2::FRT, his1::FRT/his1::FRT, CPAR2_108840::LEU2/CPAR2_108840::HIS1*	Ortholog(s) have protein kinase activity, role in activation of bipolar cell growth, ascospore wall assembly, protein phosphorylation and cell division site, cytosol, nucleus, prospore membrane localization	This study
CPAR2_200040 Δ/Δ	*leu2::FRT/leu2::FRT, his1::FRT/his1::FRT, CPAR2_200040::LEU2/CPAR2_200040::HIS1*	Ortholog(s) have nucleolus localization	This study
CPAR2_200390 Δ/Δ	*leu2::FRT/leu2::FRT, his1::FRT/his1::FRT, CPAR2_200390::LEU2/CPAR2_200390::HIS1*	Ortholog in *C. albicans* is a functional homolog of S. cerevisiae Spt3p; required for virulence in mouse systemic infection; homozygous null mutant is hyperfilamentous	This study

Strain name	Genotype	Putative function	Reference
CPAR2_202040 Δ/Δ	*leu2::FRT/leu2::FRT, his1::FRT/his1::FRT, CPAR2_202040::LEU2/CPAR2_202040::HIS1*	Ortholog(s) have RNA polymerase II core promoter proximal region sequence-specific DNA binding	This study
CPAR2_209520 Δ/Δ	*leu2::FRT/leu2::FRT, his1::FRT/his1::FRT, CPAR2_209520::LEU2/CPAR2_209520::HIS1*	Ortholog(s) have MAP kinase kinase activity, structural constituent of cell wall activity	This study
CPAR2_300080 Δ/Δ	*leu2::FRT/leu2::FRT, his1::FRT/his1::FRT, CPAR2_300080::LEU2/CPAR2_300080::HIS1*	Ortholog(s) have adenylate kinase activity, nucleoside triphosphate adenylate kinase activity, role in nucleotide metabolic process and mitochondrial inner membrane localization	This study
CPAR2_302400 Δ/Δ	*leu2::FRT/leu2::FRT, his1::FRT/his1::FRT, CPAR2_302400::LEU2/CPAR2_302400::HIS1*	Ortholog(s) have palmitoyltransferase activity, role in protein palmitoylation and plasma membrane localization	This study
CPAR2_303700 Δ/Δ	*leu2::FRT/leu2::FRT, his1::FRT/his1::FRT, CPAR2_303700::LEU2/CPAR2_303700::HIS1*	Ortholog(s) have role in positive regulation of transcription from RNA polymerase II promoter, telomere maintenance and EKC/KEOPS complex, cytosol, nucleus localization	This study
CPAR2_304080 Δ/Δ	*leu2::FRT/leu2::FRT, his1::FRT/his1::FRT, CPAR2_304080:LEU2/CPAR2_304080::HIS1*	Ortholog(s) have adenylylsulfate kinase activity	This study
CPAR2_400270 Δ/Δ	*leu2::FRT/leu2::FRT, his1::FRT/his1::FRT, CPAR2_400270::LEU2/CPAR2_400270::HIS1*	Ortholog(s) have cytoplasm, nucleus localization	This study
CPAR2_401150 Δ/Δ	*leu2::FRT/leu2::FRT, his1::FRT/his1::FRT, CPAR2_401150::LEU2/CPAR2_401150::HIS1*	Ortholog(s) have ATP binding, ATPase activity, dinucleotide insertion or deletion binding, loop DNA binding, single-stranded DNA binding activity	This study

Strain name	Genotype	Putative function	Reference
CPAR2_500180 Δ/Δ	*leu2::FRT/leu2::FRT, his1::FRT/his1::FRT, CPAR2_500180::LEU2/CPAR2_500180::HIS1*	Ortholog(s) have protein kinase activity	This study
CPAR2_501400 Δ/Δ	*leu2::FRT/leu2::FRT, his1::FRT/his1::FRT, CPAR2_501400::LEU2/CPAR2_501400::HIS1*	Ortholog(s) have mannosyl-oligosaccharide glucosidase activity	
CPAR2_502720 Δ/Δ	*leu2::FRT/leu2::FRT, his1::FRT/his1::FRT, CPAR2_502720::LEU2/CPAR2_502720::HIS1*	Ortholog(s) have protein kinase activity, role in DNA damage checkpoint, protein phosphorylation, regulation of circadian rhythm, replication fork protection and nucleus localization	This study
CPAR2_602430 Δ/Δ	*leu2::FRT/leu2::FRT, his1::FRT/his1::FRT, CPAR2_602430::LEU2/CPAR2_602430::HIS1*	Ortholog(s) have chaperone binding activity, role in ER-associated ubiquitin-dependent protein catabolic process, protein folding in endoplasmic reticulum, response to unfolded protein and endoplasmic reticulum lumen localization	This study
CPAR2_602820 Δ/Δ	*leu2::FRT/leu2::FRT, his1::FRT/his1::FRT, CPAR2_602820::LEU2/CPAR2_602820::HIS1*	Ortholog(s) have cytosine deaminase activity, role in cytidine metabolic process, cytosine metabolic process, pyrimidine-containing compound salvage and cytosol, nucleus localization	This study
